# Inhibition of SRC prevents bone metastasis of breast cancer by blocking metastatic cell motility and bone directionality

**DOI:** 10.7150/thno.130647

**Published:** 2026-05-18

**Authors:** Yong June Choi, Minju Kwon, Myung Jun Kim, Munkyung Choi, Phuong Thao Tran, Yujeong Lee, Wan Seob Shim, Minjae Kang, Seungseok Oh, Sung-Chul Lim, Yong-Chul Kim, Keon Wook Kang

**Affiliations:** 1 College of Pharmacy, Research Institute of Pharmaceutical Sciences and Natural Product Research Institute, Seoul National University, Seoul, 08826, Republic of Korea.; 2 Center for Cancer Immunology, Krantz Family Center for Cancer Research, Massachusetts General Hospital and Harvard Medical School, Boston, MA, 02129, USA.; 3 Department of Pathology, School of Medicine, Chosun University, Gwangju, 61452, Republic of Korea.; 4 School of Life Sciences, Gwangju Institute of Science and Technology, Gwangju, 61005, Republic of Korea.

**Keywords:** breast cancer bone metastasis, SRC signaling, scaffolding function, next-generation SRC inhibitor, NXP900

## Abstract

**Rationale:**

Breast cancer bone metastasis remains a major cause of mortality with limited effective therapies. Although SRC is one of the earliest identified oncogenic kinases and has been extensively studied as a regulator of cancer progression and migration, it has not yet been successfully translated into an effective therapeutic target in solid tumors, highlighting the need to redefine SRC-targeted strategies in this context.

**Methods:**

Genetic deletion and pharmacological approaches were employed to interrogate SRC function, including comparative evaluation of conventional kinase inhibitors and next-generation inhibitors targeting both kinase and scaffolding functions. Cytoskeletal remodeling and cell motility were assessed via F-actin organization and focal adhesion signaling. Preclinical bone metastasis models were used to assess the extent of bone metastasis. Therapeutic efficacy was evaluated under both monotherapy and combination regimens with gemcitabine/bisphosphonate or anti–PD-1.

**Results:**

SRC phosphorylation regulated the activation of cytoskeletal regulators FAK and paxillin, thereby controlling cancer cell motility. Genetic deletion or pharmacological inhibition of SRC significantly suppressed cell motility, reduced F-actin remodeling, increased bone density, and inhibited bone metastasis *in vivo*. SRC inhibition also attenuated immune evasion, enhanced anti-tumor immunity, and synergized with anti–PD-1 or gemcitabine/bisphosphonate therapies. Notably, a next-generation SRC inhibitor that targets both catalytic activity and scaffolding function achieved more potent suppression of metastatic signaling and bone metastasis than conventional SRC inhibitors.

**Conclusions:**

This study demonstrates that SRC plays distinct and essential roles in cancer cell motility, osteoclast activation, and immune evasion, which collectively drive breast cancer bone metastasis. These findings establish SRC as a critical therapeutic target and suggest that dual inhibition of its kinase and scaffolding functions represents a more effective strategy than conventional approaches.

## Introduction

Breast cancer is the most commonly diagnosed malignancy among women worldwide, accounting for approximately 30% of all newly diagnosed cancers in women each year 1. While early-stage breast cancer can often be effectively treated with surgery and radiation therapy, more than 20% of patients eventually develop metastatic disease 2. Although breast cancer can spread to various organs, bone is the most common site of metastasis 3. Bone metastases frequently lead to severe complications such as fractures and spinal cord compression, significantly impairing overall survival rates 3, 4. More than 80% of breast cancer cases occur in women over the age of 50 5, and menopause-associated bone loss or osteoporosis has been linked to increased susceptibility to bone metastasis and poor prognosis in breast cancer 6, 7. Thus, bone-protective medications, such as bisphosphonates and receptor activator of nuclear factor kappa-Β ligand (RANKL) inhibitors (e.g., denosumab), are often used to prevent or manage bone metastases 8, 9. Nonetheless, to date there are no effective treatment options for bone metastases, highlighting the urgent need for new therapeutic strategies 10.

In primary tumors, cancer cells initiate metastasis by increasing their cell motility and reducing adhesion 11. They spread to other organs via blood vessels, lymphatic vessels, or direct organ-to-organ migration 12. Although disseminated cancer cells with enhanced motility may reach multiple organs, metastatic outgrowth preferentially occurs in microenvironments that support tumor cell survival and reactivation, as explained by the "seed and soil" hypothesis 13. This theory suggests that certain organs or pathological conditions create favorable environments for cancer colonization and reactivation. For instance, pulmonary fibrosis creates a niche that support the survival of metastatic cancer cells in the lungs 14, while metabolic dysfunction-associated steatotic liver disease (MASLD) facilitates the rapid proliferation of metastatic cancer cells in the liver 15. Thus, metastasis is a highly complex, multi-step process governed by both intrinsic pathways within cancer cells and extrinsic factors derived from the tumor microenvironment. Nonetheless, current pharmacological treatments for metastatic breast cancer predominantly focus on inhibiting cancer cell growth and motility. Therapies such as HER2-targeted agents, CDK4/6 inhibitors, poly (ADP-ribose) polymerase (PARP) inhibitors, and combination chemotherapy are commonly used in clinical practice 16-18. Given the pivotal role of the tumor microenvironment in determining metastatic organotropism and reactivation, therapeutic strategies that simultaneously target both intrinsic cancer cell pathways and extrinsic tumor microenvironmental factors are urgently needed.

One of the most well-characterized mechanisms by which the bone microenvironment promotes cancer progression is the osteolytic vicious cycle 19. In this cycle, metastatic cancer cells secrete osteolytic factors such as parathyroid hormone-related protein (PTHrP), interleukin-11 (IL-11), and RANKL, which stimulate osteoclast differentiation and activation 20-22. Activated osteoclasts subsequently degrade bone matrix and release growth factors such as transforming growth factor-β (TGF-β) and insulin-like growth factors (IGFs) 23-25. These factors enhance cancer cell proliferation, survival, and further osteolytic activity, thereby perpetuating positive feedback loop that accelerates bone metastasis 23-25. Hence, pathological conditions such as postmenopausal osteoporosis, which are characterized by increased osteolytic activity 26, may facilitate to the formation of a pro-metastatic bone niche. Although bisphosphonates and RANKL inhibitors have been clinically approved to target osteoclast-mediated bone destruction during bone metastasis 27, these agents are primarily used as palliative therapies and there is no definitive evidence that they directly inhibit bone metastases 27. Consequently, there is growing interest in developing therapeutic strategies that interfere with the molecular crosstalk between cancer cells and the bone microenvironment in order to block the osteolytic vicious cycle.

SRC family kinases (SFK) are cytoplasmic non-receptor tyrosine kinases that interact with various receptor tyrosine kinases (RTKs), and phosphorylate and activate multiple substrate proteins 28. They play a crucial role in numerous pathological conditions, and notably, are well known as key regulators of cancer cell growth and have recently been implicated in fibrotic diseases 14, 29-31. Despite their well-established roles in cancer biology, the precise substrate proteins and downstream signaling pathways of SFKs in metastasis remain incompletely defined. Clinically available SFK inhibitors such as dasatinib have been evaluated in solid tumors, including metastatic breast cancer; however, their broad multi-kinase activity and insufficient suppression of metastasis-associated signaling have limited clinical efficacy 32. Mechanistically, dasatinib has been reported to preferentially suppress SRC catalytic activity by inducing dephosphorylation at tyrosine 419, a key activation site 33, 34. In contrast, NXP900, a next-generation SRC inhibitor introduced in 2021, has been reported to inhibit SRC in a conformation-selective manner, simultaneously targeting both kinase activity and scaffolding function through coordinated dephosphorylation at Y419 and phosphorylation at Y530 33, 34. However, the functional consequences of these distinct modes of SRC inhibition—particularly with respect to metastatic signaling and cancer cell motility—have not been characterized.

Although SRC family kinases have long been implicated in cancer, SRC inhibitors have not yet been successfully translated into effective therapies for solid tumors. Here, we investigated whether SRC functions as a central signaling regulator linking cancer cell–intrinsic motility, osteoclast-mediated bone remodeling, and immune regulation during breast cancer bone metastasis. We further aimed to elucidate the molecular mechanisms by which SRC modulates cytoskeletal dynamics and downstream effector signaling, and to evaluate the therapeutic potential of targeting SRC-driven pathways to disrupt the establishment and progression of bone metastases. Notably, we introduce a novel class of SRC inhibitors capable of effectively suppressing intracellular metastatic signaling, which reduced F-actin remodeling and cell motility *in vitro* and significantly inhibited bone metastasis *in vivo*.

## Materials and Methods

### Cells and mice

Details of the cell lines used and their culture conditions are provided in Table S1. MCF-7, HEK-293T, MDA-MB-231, PC-3, MG-63 and U2-OS cell lines were obtained from Korean Cell Line Bank (Seoul, Korea). The 4T1 and 4T1-luc cell lines were donated by Dr. Byun (College of Pharmacy, Seoul National University, Seoul, Korea). All cells were maintained at 37 °C in a humidified incubator with 5% CO₂.

In all animal experiments, 5- to 7-week-old male and female BALB/c mice (Raonbio, Seoul, Korea and JA BIO, Suwon, Korea) were used and maintained in the specific-pathogen-free facility at the Seoul National University (Seoul, Korea).

### Chemotaxis transwell migration assay

To evaluate the motility of cancer cells, 3,000 cells per well were seeded in an IncuCyte Clearview 96-well plate (#4582, Sartorius, Gottingen, Germany). To establish a chemoattractant gradient, 1% FBS was added to the upper well, and 10% fetal bovine serum (FBS) was added to the lower well. Inhibitors were added to both upper and lower wells at the specified concentrations. The number of migrated cells was analyzed using the IncuCyte Zoom/S3 Live Cell Analysis System (EssenBioscience, Ann Arbor, MI, USA).

### Cell proliferation assay

Cancer cells were seeded at a density of 2 × 10³ cells per well in a 96-well plate. Following drug treatment, cell confluence was monitored and analyzed using the IncuCyte Zoom Live Cell Analysis System (EssenBioscience). Finally, cell confluence was quantified as a ratio relative to the initial time point.

### Small interfering RNA transfection and plasmid overexpression

SRC knockdown was performed using Lipofectamine 2000 transfection reagent (ThermoFisher) following the manufacturer’s protocol. Lipofectamine 2000 was combined with transfection optimized medium (WELGENE, Gyeongsan, South Korea), siRNA was added to the mixture, and incubated for 20 minutes. Cells were exposed to the siRNA mixture for 24 h. Predesigned siSRC pools and negative control siRNA were purchased from Bioneer (ID: 6714 or 20779, Daejeon, South Korea). siFAK, siPXN#1, and siPXN#2 were purchased from Bioneer, and their sequences are as follows: siFAK (sense: 5’-ACACCAAAUUCGAGUACUA-3’; antisense: 5’-UAGUACUCGAAUUUGGUGU-3’);

siPXN#1 (sense: 5’-GUGUGGAGCCUUCUUUGGU-3’; antisense: 5’-ACCAAAGAAGGCUCCACAC-3’);

siPXN#2 (sense: 5’-CCCUGACGAAAGAGAAGCCUA-3’; antisense: 5’-UAGGCUUCUCUUUCGUCAGGG-3’). SRC overexpression was conducted using the same protocol as siRNA transfection with Lipofectamine 2000 transfection reagent. The various SRC overexpression vectors used are listed below.

### The construction of SRC phospho- mutant plasmid

To mimic the constitutively dephosphorylated form of the human SRC sequence [NM_005417.5], we substituted the Tyr^419^ (TAC) and Tyr^530^ (TAC) codons with Phe^419^ (TTC) and Phe^530^ (TTC), respectively. For the constitutively phosphorylated form, we replaced these codons with Glu^419^ (GAG) and Glu^530^ (GAG). By combining these substitutions in different ways, we generated three phospho-mutant SRC expression plasmids (Phe^419^ Phe^530^), (Phe^419^ Glu^530^) and (Glu^419^ Glu^530^). These plasmids, containing the modified human SRC sequence in a mammalian gene expression vector under the control of a CMV promoter, were obtained from VectorBuilder (Chicago, IL, USA).

### Phalloidin (F-actin) staining: immunofluorescence

Alexa Fluor 488 Phalloidin (#A12379, Invitrogen) was used to stain F-actin in cancer cells following the manufacturer's instructions. Confocal microscopy was used to visualize F-actin (TCS SP8, Leica, Wetzlar, Germany). The intensity of F-actin was analyzed using ImageJ (Bethesda, MD, USA). All individual F-actin images have been included in the figures or provided in the Supplementary Materials.

### Western blot

Cell lysis was performed using a buffer containing Triton X-100 (1%), sodium pyrophosphate (30 mM), sodium chloride (100 mM), glycerol (10%), EDTA (1 mM), Tris-Cl (10 mM), glycerol-2-phosphate (5 mM), sodium orthovanadate (1 mM), sodium fluoride (1 mM), phosphatase inhibitor cocktail 2 (1%, Sigma-Aldrich, St. Louis, MO, USA), protease inhibitor cocktail (0.02 tablet/mL, Roche, Basel, Switzerland), and phosphatase inhibitor cocktail 3 (1%, Sigma-Aldrich). Protein concentrations were determined using the Bradford assay. The samples were then separated by SDS-PAGE and transferred onto 0.45 μm nitrocellulose membranes (Cytiva, Marlborough, MA, USA). Membranes were blocked with 5% skim milk in phosphate-buffered saline containing 0.1% Tween 20 (PBST) and incubated with primary antibodies overnight at 4 °C, followed by incubation with secondary antibodies for 1 h at room temperature. Signals were visualized using a chemiluminescent HRP substrate and detected with the ImageQuant LAS-4000 (GE Healthcare, Chicago, IL, USA). Antibodies used in this study are listed in Table S2. All western blot experiments were conducted independently at least three times, with all immunoblot images provided in the Supplementary Materials.

### Bone metastasis and lung metastasis mouse model

To assess the extent of bone metastasis, 4T1-luc cells (5 × 10^4^ cells) were injected into the tail artery of mice. In the lung metastasis model, 4T1-luc cells (5 × 10^5^ cells) were injected into the tail vein of mice. Luminescence intensity in the bone was measured using the IVIS Spectrum (PerkinElmer, Waltham, MA, USA). For luminescence detection, 150 mg/kg D-Luciferin Potassium Salt (PerkinElmer) was administered via intraperitoneal injection 10 minutes prior to imaging. Mice with impaired ambulation in the lower extremities due to bone metastases were euthanized following ethical guidelines, and survival rates were documented. These procedures were approved by the Institutional Animal Care and Use Committee (IACUC) of Seoul National University (Approval #SNU-230709-1, SNU-230830-1, SNU-231221-1).

### Ovariectomy (OVX) mouse model

To replicate menopause-associated bone loss, ovariectomy was performed on 5- to 7-week-old female BALB/c mice. Following anesthesia, an incision was made in the abdomen to remove both ovaries, after which an antibiotic ointment was applied to the surgical site until complete wound healing. All wounds fully healed within one week post-ovariectomy, and a significant reduction in bone density was observed after four weeks. These procedures were approved by the IACUC of Seoul National University (Approval #SNU-231209-1, SNU-240522-3).

### Flank allograft mouse model

4T1 mouse breast cancer cells (1 × 10^6^ cells) were subcutaneously injected into the flanks of mice. Once tumors became palpable (100-150 mm^3^), drug treatments were administered over the specified days. Tumor volume and body weight were measured twice a week. Tumor volume was calculated using a caliper as followed formula: length × (width)^2^ × 0.5. These procedures were approved by the IACUC of Seoul National University (Approval #SNU-230402-1).

### *In vivo* treatment

To assess the efficacy of SRC-targeted drugs in animal models, mice received Dasatinib or NXP900 (60 mg/kg, diluted in 3 mM sodium citrate buffer) once daily via oral gavage for the specified duration. For evaluating combination therapy with current breast cancer treatments, Gemcitabine was administered at 30 mg/kg by intraperitoneal injection twice weekly, and Zoledronate at 0.2 mg/kg by intraperitoneal injection once weekly (Gem/BP). Anti-mouse PD-1 antibody was administered at 10 mg/kg by intraperitoneal injection twice a week. The drug sources are detailed in Table S3.

### Evaluation of bone loss: micro-CT, flow cytometer, and immunohistochemistry (IHC) analysis

The extent of bone resorption was assessed using the Micro-CT (PerkinElmer). Mouse bone density was subsequently analyzed with Micro-CT analysis software (Caliper LifeSciences, Hopkinton, MA, USA) and ImageJ software.

For flow cytometer analysis, mouse bone marrow cells were isolated and stained with MMP9 antibodies. The antibodies used for flow cytometry are listed in Table S2.

For IHC analysis, bone tissues were dissected from sacrificed mice, embedded in paraffin blocks, and stained with Hematoxylin and Eosin (H&E). Stained slides were imaged using a Vectra instrument (PerkinElmer) and analyzed with ImageJ software. These procedures were approved by the IACUC of Seoul National University (Approval #SNU-231209-1, SNU-240522-3).

### Isolation and differentiation of primary osteoclast and macrophage

Following trimming of the femur and tibia, bone marrow cells were harvested. Red blood cells were lysed using ACK lysis buffer (Gibco, Gaithersburg, MD, USA). For differentiation to osteoclast, cells were seeded in petri dish and cultured with 30 ng/mL macrophage-colony stimulating factor (M-CSF) for 3 days. Following this, 30 ng/mL M-CSF and 100 ng/mL receptor activator of nuclear factor kappa-B ligand (RANKL, Enzynomics, Daejeon, Korea) were added for an additional 6 days. The differentiated primary osteoclasts were subsequently used in further experiments. For differentiation to macrophage, bone marrow cells were treated with 30 ng/mL M-CSF for 6 days. These procedures were approved by the IACUC of Seoul National University (Approval #SNU-230830-2, SNU-241105-8).

### Growth factor array

Monocytes and activated osteoclasts were incubated in serum-free RPMI 1640 (with HEPES) for 15 h, and each collected supernatant with secreted growth factors was applied to the Mouse Growth Factor Array C3 kit (Cat. No. AAM-GF-3-4, Raybiotech, GA, USA) following the manufacturer’s instructions. Briefly, samples were incubated on an array chip coated with antibodies specific to 30 distinct growth factors. Detection was subsequently performed using biotinylated secondary antibodies and labeled streptavidin, enabling quantitative comparison of the relative concentrations of each growth factor in the samples. A positive control was utilized to normalize the signal intensities across different membranes for accurate comparison.

### Enzyme-linked immunosorbent assay (ELISA)

Activated osteoclasts pre-treated with dasatinib, NXP900, or vehicle were incubated in fresh serum-free RPMI 1640 (with HEPES) for 15 h, and each collected supernatant with secreted IGF-1 was applied to Quantikine ELISA Mouse/Rat IGF-1 Immunoassay kit (Cat. No. MG100, R&D Systems, MN, USA) following the manufacturer’s instructions. Briefly, ELISA was conducted using a quantitative sandwich enzyme immunoassay technique. Samples were incubated on a microplate pre-coated with a monoclonal antibody specific for mouse/rat IGF-1 and the amount of IGF-1 in each sample was determined by measuring the color intensity, which is directly proportional to the IGF-1 concentration. ELISA was performed on all samples in triplicate to ensure consistency.

### Immune cell analysis

Tumor tissue, inguinal lymph nodes, and spleen were collected for flow cytometry analysis. The tissues were dissociated by gentleMACS dissociator and a dissociation kit (Miltenyi Biotec, Bergisch Gladbach, Germany). Immune cells were subsequently enriched from dissociated tissues using a Percoll density gradient, followed by staining with antibodies. Flow cytometry analysis was performed using a Novocyte flow cytometer (Agilent, Santa Clara, CA, USA). The antibodies used for flow cytometry are listed in Table S2. These procedures were approved by the IACUC of Seoul National University (Approval #SNU-230402-1, SNU-241105-7).

### Adoptive transfer of CD8+ T cells

4T1 mouse breast cancer cells (1 × 10^6^ cells) were subcutaneously injected into the flanks of mice. Once tumors became palpable (100-150 mm^3^), drug treatments were administered over the specified days. Inguinal lymph nodes and spleen were collected for CD8 T cell isolation. After dissociating the tissue, immune cells were isolated using a percoll gradient, followed by attachment of mouse CD8 microbeads (#130-117-044, Miltenyi Biotec). The CD8+ cells were then separated using a MACS LS column (Miltenyi Biotec) with a magnet. The isolated CD8 T cells (2 × 10^5^ cells) were injected into new mice with induced bone metastasis (on day 0) at two time points: days -2 and 5. To verify that the transferred CD8+ T cells specifically inhibited bone metastasis of 4T1 cancer cells, the CD8+ T cells were labeled with Vivotrack680 dye (#NEV12001, PerkinElmer), introduced into mice with either 4T1 flank tumors or bone metastases, and then analyzed. These procedures were approved by the IACUC of Seoul National University (Approval #SNU-230402-1, SNU-241105-7).

### Public data

Using the Gene Expression Profiling Interactive Analysis (GEPIA) web server, survival analysis based on gene expression in tumor patients was performed 35. The CancerHallmarks database was utilized to examine cancer hallmark gene sets associated with specific gene groups 36. Kaplan-Meier Plotter 37 and ROC Plotter 38 databases were employed to analyze gene expression and survival rates in patients with or without anti-PD-1 treatment. Patient sample analyses were performed using data from the NCBI GEO database, specifically GSE27574 39 and GSE230665 40. Patient data on bone metastasis in breast and prostate cancer were obtained from the Metastatic Breast Cancer Project 41 (https://www.mbcproject.org/), the Metastatic Prostate Cancer Project (http://www.mpcproject.org/), and a project of Count Me In (https://joincountmein.org/). Physical and functional interactions of SRC were analyzed using the STRING database 42.

### Statistical analysis

Statistical significance was evaluated using GraphPad Prism 7.0 (GraphPad, Boston, MA, USA). Differences between groups were assessed using either one-way ANOVA followed by Tukey's test or an unpaired two-tailed Student's t-test. P values below 0.05 were considered statistically significant. In this study, p values are presented in NEJM style as follows: *p < 0.05, **p < 0.01, ***p < 0.001.

## Results

### SRC is associated with bone metastasis of breast cancer

**Figure 1:**
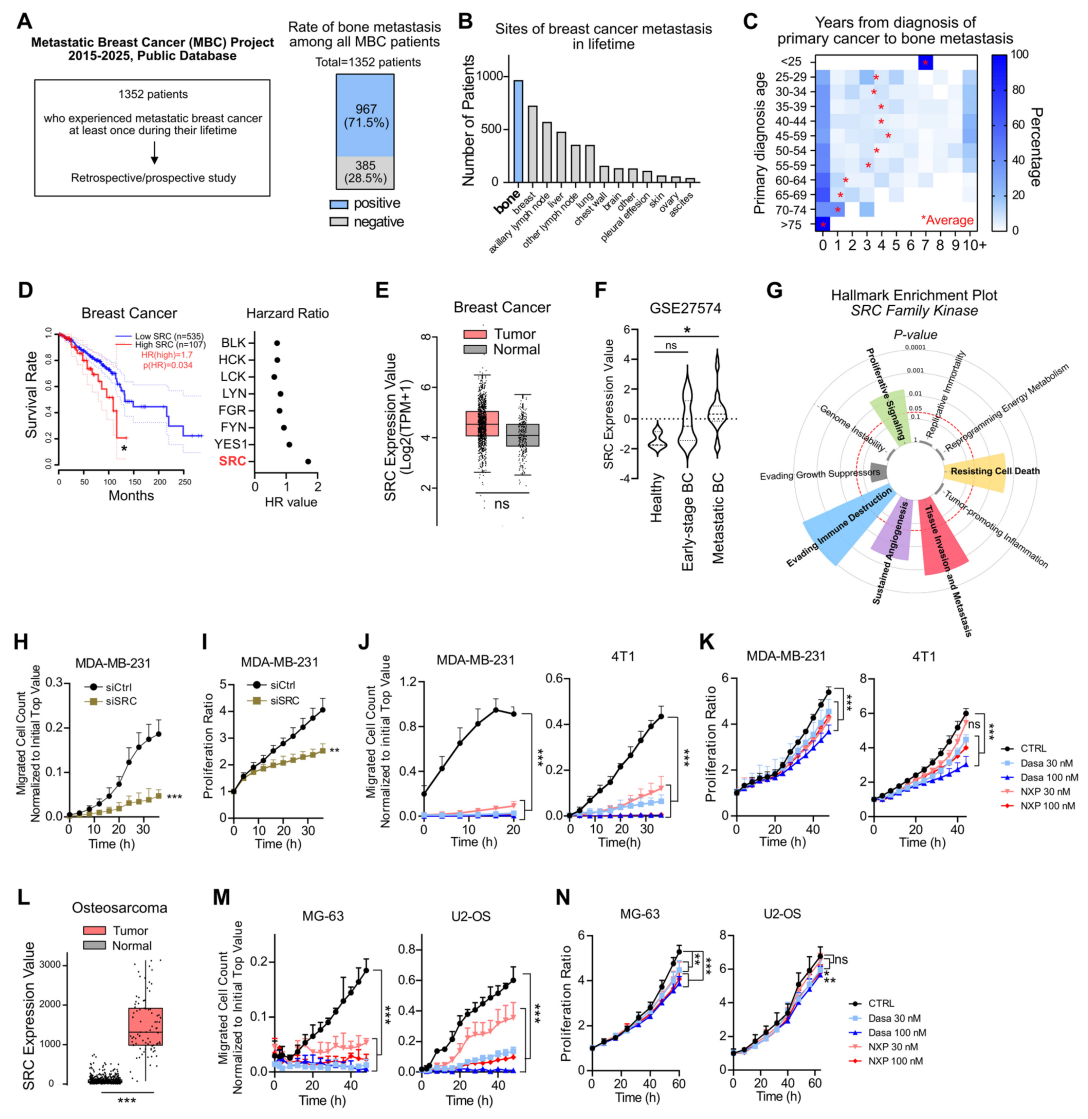
**SRC is linked to breast cancer bone metastasis and poor patient outcomes.** (A, B) Bone metastasis cases in metastatic breast cancer patients were analyzed through both prospective and retrospective investigations [The Metastatic Breast Cancer (MBC) Project, https://www.mbcproject.org/]. (C) Interval time from primary tumor diagnosis to metastatic diagnosis. The mean diagnostic interval for each age group is indicated. (The MBC Project). (D) Survival rate of breast cancer patients according to SRC expression (left) and hazard ratios of SRC family kinases (right). Data were obtained from the GEPIA public database. (E) Comparison of SRC expression between breast cancer and normal tissues using the GEPIA database. (F) Comparison of SRC expression in disseminated tumor cells between early-stage and metastatic breast cancer patients. “Healthy” indicates hematopoietic cells from normal donors. Dataset: GSE27574. (G) Cancer hallmark pathways associated with SRC family kinases. Bar length indicates significance (–log p-value). Analysis was performed using the Cancer Hallmarks database. (H, I) Changes in transwell migration and proliferation of metastatic breast cancer cells following SRC knockdown. (J, K) Effects of SRC inhibitors on transwell migration and proliferation of metastatic breast cancer cells. (L) Comparison of SRC expression between osteosarcoma and normal tissues using the TNMplot database. (M, N) Effects of SRC inhibitors on transwell migration and proliferation of osteosarcoma cells. All data are presented as mean with standard deviation. Statistical significance was determined by two-tailed Student’s t-test or one-way ANOVA followed by Tukey’s test. *p < 0.05, **p < 0.01, ***p < 0.001, significant difference between indicated points.

Metastasis is directly associated with the overall survival of cancer patients 11, 18. We analyzed publicly available datasets of patients with metastatic breast cancer collected between 2015 and 2025 41. Among 1,352 breast cancer patients who experienced at least one metastatic event, 71.5% developed bone metastasis (Figure 1A), confirming bone as the most frequent site of metastasis (Figure 1B). The interval between primary breast cancer diagnosis and the onset of bone metastasis was significantly shorter in patients older than 50 years of age (Figure 1C). Specific breast cancer subtypes exhibit distinct metastatic patterns 43. In line with previous findings, our analysis of public datasets revealed that patients with bone metastasis had a higher proportion of ER/PR-positive tumors and a lower proportion of TNBC compared with those without bone metastasis (Figure S1A-B). Nevertheless, bone metastasis was observed across all breast cancer subtypes, including TNBC (Figure S1A-B). In subtype-specific survival analyses, high SRC expression was associated with poorer patient survival across all breast cancer subtypes except luminal B (Figure S1C). Women experience postmenopausal bone loss after the age of 50, with progressive decline in bone mineral density during aging 6, 26. Because metastasis is determined not only by the migratory ability of cancer cells but also by microenvironmental alterations at metastatic sites, we focused on molecular targets implicated in both osteoporosis and breast cancer progression. Elevated SRC expression was associated with poorer survival in breast cancer patients (Figure 1D). Interestingly, SRC expression was not significantly increased in primary breast cancer tissues compared with normal tissues (Figure 1E); however, it was significantly elevated in patients with metastatic breast cancer (Figure 1F). These findings led us to hypothesize that SRC play a key role in promoting bone metastasis and thereby contributes to reduced survival in breast cancer patients.

We further examined the biological hallmarks of cancer associated with SRC expression, and found that SRC was significantly linked to proliferation, resistance to cell death, metastasis, angiogenesis, and immune evasion (Figure 1G). Knockdown of SRC in human breast cancer cells markedly reduced cell migration and partially inhibited proliferation (Figure 1H-I). To further validate the effects of SRC inhibition in breast cancer, we used two SRC inhibitors, dasatinib and NXP900. Both inhibitors potently suppressed cancer cell migration, while partially reduced cell proliferation (Figure 1J-K).

Prostate cancer is one of the most common malignancies in men that metastasizes to bone 3. Approximately 50% of patients with metastatic prostate cancer develop bone metastases (Figure S2A). Consistently, SRC expression was associated with poor survival in prostate cancer patients (Figure S2B), and treatment with SRC inhibitors significantly suppressed the migratory ability of human prostate cancer cells (Figure S2C-D).

Although originating from different tissues, bone-metastatic breast cancer cells and primary bone tumors share common dependencies on the reciprocal interactions with the bone microenvironment for their growth and survival 3, 44. Therefore, we further examined the functional role of SRC using osteosarcoma model. In osteosarcoma tissues, SRC expression was significantly higher than in normal tissue, resembling the expression pattern observed in metastatic breast cancer (Figure 1L). Consistently, both SRC inhibitors significantly suppressed the migration of human osteosarcoma cells and partially reduced cell proliferation (Figure 1M-N). Collectively, these findings demonstrate that SRC plays a pivotal role in promoting breast cancer bone metastasis and contributes to tumor progression within the bone microenvironment.

### SRC regulates cancer cell motility through regulation of F-actin dynamics

**Figure 2:**
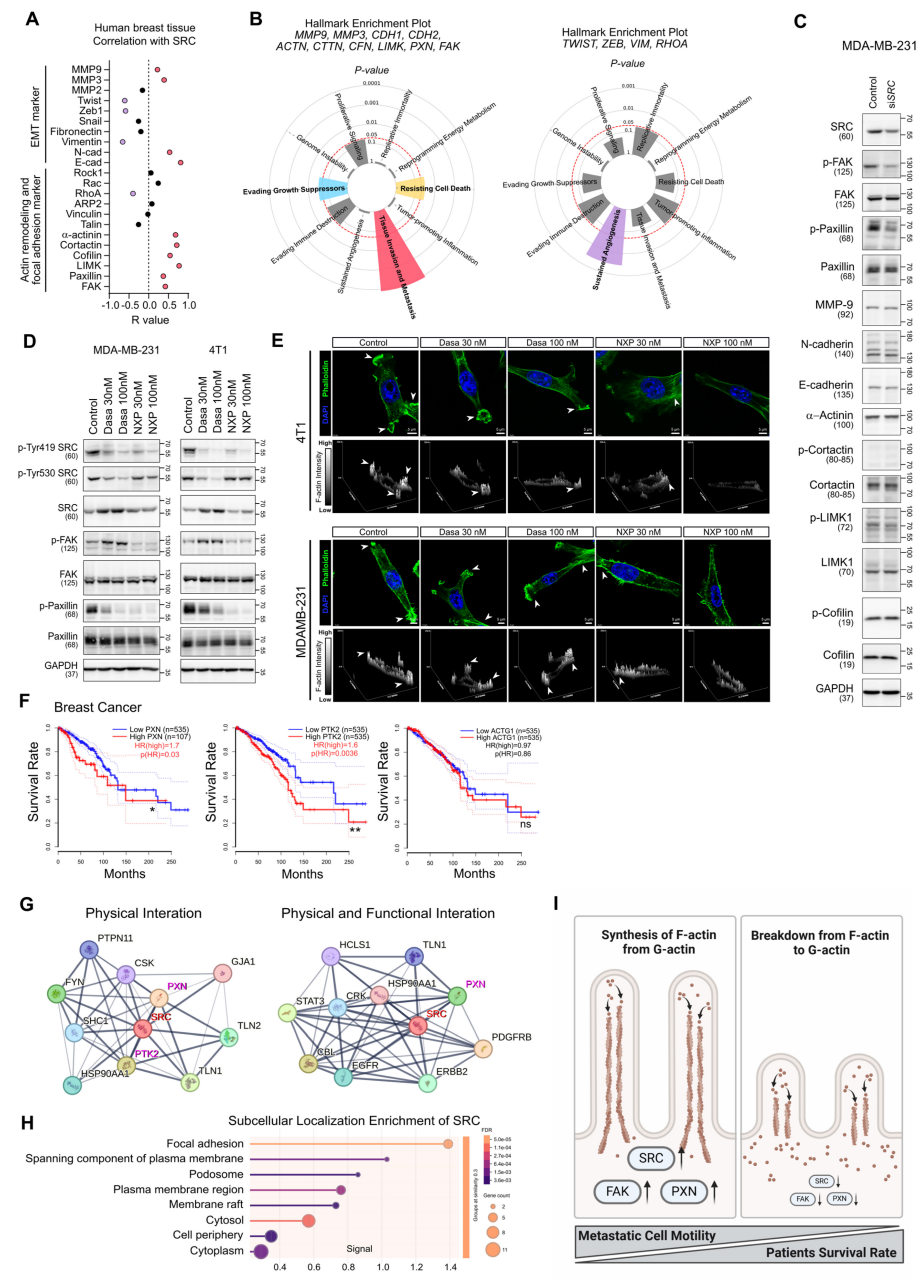
** SRC regulates cancer cell motility by modulating paxillin-F-actin dynamics.** (A) Correlation analysis between SRC expression and cell motility–related genes in human breast tissue. Data were obtained from the GEPIA public database. (B) Cancer hallmark pathways associated with the indicated gene clusters. Left, genes positively correlated with SRC expression; right, genes negatively correlated with SRC expression. Analysis was performed using the Cancer Hallmarks database. (C) Changes in the activation of cell motility–related proteins following SRC knockdown. (D) Changes in SRC, FAK, and paxillin activation in breast cancer cells following treatment with SRC inhibitors. (E) Representative confocal images of breast cancer cells treated with vehicle or SRC inhibitors, showing F-actin–rich invadopodia structures. Cells were stained with phalloidin (green) to visualize F-actin and DAPI (blue) to label nuclei. Arrowheads indicate invadopodia-like actin puncta. Z-stack reconstruction with orthogonal projections and surface plots is shown to visualize the spatial organization, vertical distribution, and relative intensity of F-actin structures in control and SRC inhibitor–treated cells. (F) Survival analysis of breast cancer patients based on the expression of PXN (paxillin), PTK2 (FAK), and ACTG1 (G-actin). Data were obtained from the GEPIA database. (G) Analysis of the top ten proteins interacting with SRC. Left, physical interactions; right, physical and functional interactions. Data were obtained from the STRING database. (H) Subcellular localization enrichment analysis of SRC using the STRING database. (I) Schematic illustration depicting SRC-mediated regulation of paxillin and F-actin dynamics driving metastatic motility. Statistical significance of the differences was determined by two-tailed Student’s t-test. *p < 0.05, **p < 0.01, ***p < 0.001, significant difference between indicated points.

Next, we investigated the molecular mechanism by which SRC regulates cancer cell motility. Correlation analysis of SRC expression with motility-related marker genes in human breast tissues was performed (Figure 2A). Genes positively correlated with SRC expression were strongly associated with cancer metastasis, whereas negatively correlated genes were linked to angiogenesis (Figure 2B). In breast cancer cells, SRC knockdown selectively reduced the activation of FAK and paxillin, key regulators of actin cytoskeletal remodeling (Figure 2C). Similarly, treatment with two SRC inhibitors commonly suppressed the activation of paxillin and consequently inhibited F-actin assembly within invadopodia of cancer cells (Figure 2D-E). These effects were consistently observed in both prostate cancer and osteosarcoma cells (Figure S2E-G).

Breast cancer bone metastasis was most prevalent in the ER/PR-positive subtype, and high SRC expression was most strongly associated with worse survival in the luminal A subtype (ER+/PR+/HER2-) among all subtypes (Figure S1C). Accordingly, we evaluated SRC function using an ER-positive human breast cancer cell line, MCF7. Although MCF7 lacks migratory capacity *in vitro*, precluding direct assessment of the anti-metastatic effects of SRC inhibitors, these inhibitors suppressed paxillin activation and consequently inhibited F-actin assembly in cancer cells (Figure S3).

Dasatinib inhibits multiple kinases, including SRC, BCR-ABL, c-KIT, and PDGFRβ, and therefore its effects may reflect SRC-non-specific activity 45. To address this, we additionally evaluated Saracatinib, a SRC and ABL inhibitor 45. Consistent with previous results, Saracatinib exerted a more pronounced inhibitory effect on migration than on proliferation (Figure S4A-B). It also suppressed paxillin activation and inhibited F-actin assembly within invadopodia (Figure S4C-D). These findings indicate that SRC inhibitors suppress cancer cell motility through the paxillin–F-actin axis.

Next, we assessed the roles of paxillin and FAK, key components of the focal adhesion complex downstream of SRC, in regulating F-actin–rich invadopodia-dependent cell motility. Knockdown of paxillin in breast cancer cells markedly reduced cell migration and partially impaired proliferation, similar to the effects observed with SRC inhibition (Figure S5A-C). Paxillin knockdown also inhibited F-actin formation in invadopodia (Figure S5D). Treatment with Cytochalasin D, an inhibitor of actin polymerization, similarly suppressed F-actin assembly and reduced the motility of breast cancer cells (Figure S5E-G). In the same cells, FAK knockdown similarly exerted a stronger inhibitory effect on cell migration than on cell proliferation and suppressed F-actin formation in invadopodia (Figure S6). Knockdown of paxillin and FAK mutually suppressed each other’s activation, indicating that these proteins function cooperatively in regulating F-actin formation (Figure S5C and Figure S6C).

Both FAK and paxillin expression were associated with poor survival in breast cancer patients, whereas increased expression of ACTG1, which encodes G-actin, had no significant impact on patient survival (Figure 2F). SRC physically and functionally interacted with FAK and paxillin (Figure 2G), and localized to cellular structures directly involved in cell motility, including focal adhesions, plasma membrane–spanning components, and podosomes (Figure 2H). Collectively, our findings reveal that SRC-mediated activation of focal adhesion complex and the subsequent G-actin-to-F-actin transition are critical determinants of breast cancer cell motility and survival in breast cancer patients (Figure 2I).

### SRC activation states govern differential downstream signaling

SRC is known to be regulated by the phosphorylation status at two key tyrosine residues: phosphorylation at Y419 promotes its kinase activity, whereas phosphorylation at Y530 inhibits its scaffolding function 46. Dasatinib and Saracatinib selectively suppress SRC catalytic activity by inducing dephosphorylation at Y419 33, 34. In contrast, NXP900, a next-generation SRC inhibitor, targets both kinase activity and scaffolding function by inducing dephosphorylation at Y419 and phosphorylation at Y530 33, 34. However, downstream substrate activation and consequences of these distinct SRC phosphorylation states has not been fully characterized.

Consistent with previous findings 33, 34, our results demonstrated that the two SRC inhibitors differentially modulated SRC phosphorylation at Y419 and Y530 in both MDA-MB-231, MCF7 and 4T1 cells, leading to distinct SRC activation states (Figure 2D and Figure S3B). To further investigate how these altered SRC activation states affect downstream signaling, we focused on differential regulation of FAK activity (Figure 2D, Figure S2E-F, Figure S3B and Figure S4C). Compensatory activation of FAK following dasatinib or Saracatinib treatment limited the complete suppression of paxillin signaling and subsequent F-actin assembly, whereas NXP900 completely abrogated downstream signaling (Figure 2D-E, Figure S2E-G, Figure S3B-C and Figure S4C-D).

**Figure 3:**
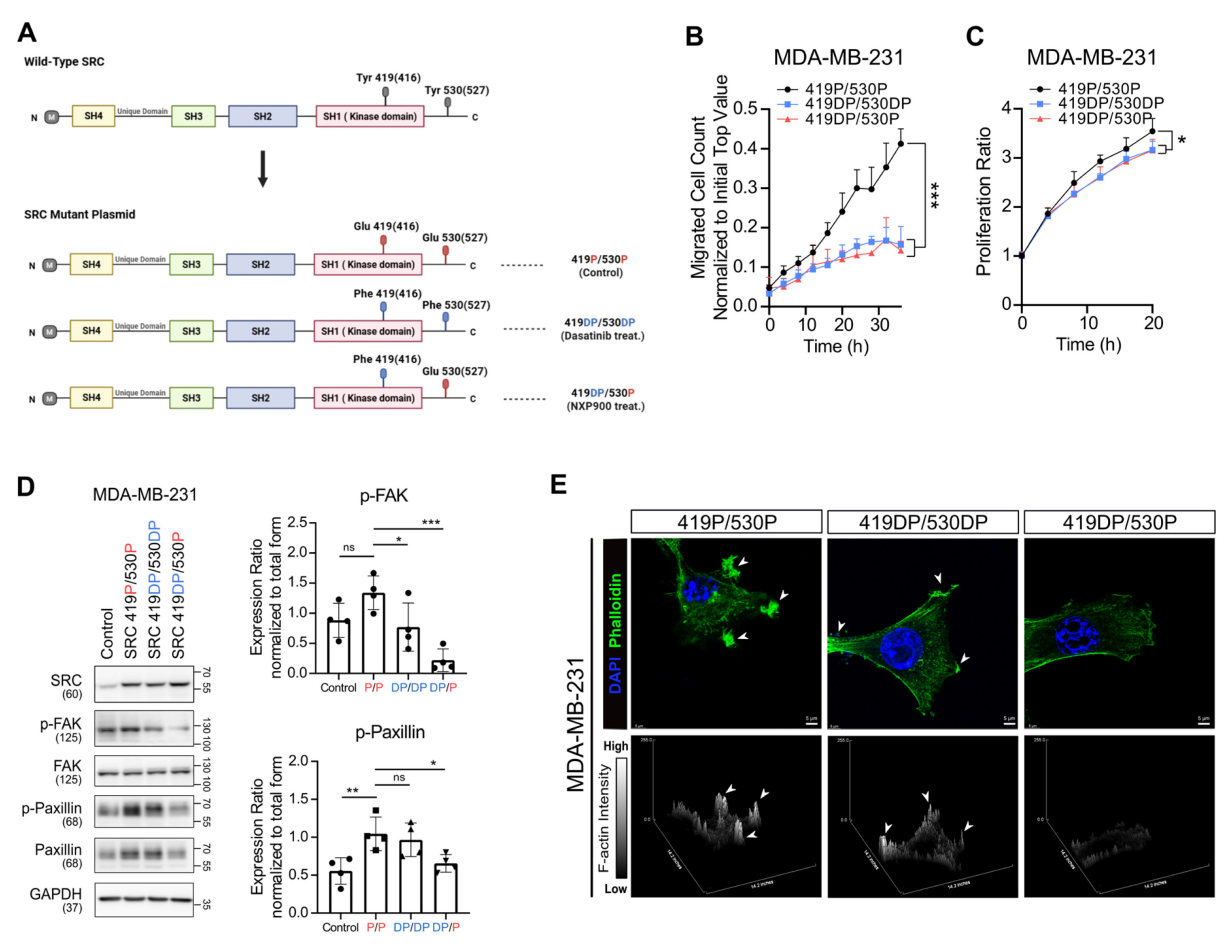
** Phosphorylation-dependent SRC activation governs distinct downstream signaling and F-actin dynamics.** (A) Schematic diagram of phospho-mutant SRC plasmid constructs used to dissect phosphorylation-dependent SRC activity. (B, C) Changes in transwell migration and proliferation of breast cancer cells following phospho-mutant SRC plasmids overexpression. (D) Changes in activation of FAK and paxillin after overexpression of phospho-mutant SRC plasmids. (E) Representative confocal images of MDA-MB-231 breast cancer cells expressing SRC phospho-mutant constructs (419P/530P, 419DP/530DP, and 419DP/530P), showing F-actin–rich invadopodia-like structures. Cells were stained with phalloidin (green) to visualize F-actin and DAPI (blue) to label nuclei. Arrowheads indicate invadopodia-like actin puncta. Z-stack images were reconstructed and displayed as surface plots to visualize the spatial organization, vertical distribution, and relative intensity of F-actin signals across the indicated SRC phospho-mutant conditions. All data are presented as mean with standard deviation. Statistical significance was determined by two-tailed Student’s t-test or one-way ANOVA followed by Tukey’s test. *p < 0.05, **p < 0.01, ***p < 0.001, significant difference between indicated points.

To further clarify SRC activation state-dependent signaling, we generated a series of phospho-mutant SRC constructs in which tyrosine residues of SRC were substituted with glutamic acid (Glu, phosphomimetic) or phenylalanine (Phe, dephosphomimetic) (Figure 3A). Although phosphorylation at Y419 and Y530 exerts opposing results in terms of SRC activity, these modifications are not mutually exclusive. In fact, in most human breast cancer tissues, SRC is found to be dually phosphorylated at Y419 and Y530 (Y419P/Y530P) 47, similar to what we observed across multiple cancer cell lines (Figure 2D, Figure S2E-F and Figure S3B). Nevertheless, the Y419P/Y530DP state may represent a maximally active form of SRC. Therefore, we initially used the corresponding mutant constructs. Interestingly, the Y419P/Y530DP mutant did not further enhance downstream SRC signaling or metastatic potential compared with the Y419P/Y530P mutant (Figure S7).

Then, these three phospho-mutant SRC constructs (419P/530P, 419DP/530DP, and 419DP/530P) were individually overexpressed in MDA-MB-231 cells. Transwell migration and proliferation assays showed that suppression of SRC catalytic activity (Y419DP) reduced cell migration and proliferation (Figure 3B-C), whereas inhibition of SRC scaffolding function (Y530P) resulted in more pronounced suppression of FAK and paxillin activation and F-actin assembly (Figure 3D-E). Collectively, these findings indicate that distinct SRC phosphorylation states differentially regulate downstream signaling pathways, and suggest that SRC inhibitors capable of inhibiting scaffolding function, similar to NXP900, may more effectively suppress breast cancer cell motility by achieving maximal inhibition of SRC–FAK/paxillin signaling, whereas conventional SRC inhibitors fail to fully suppress this pathway due to compensatory FAK activation.

### SRC promotes osteoclast activation and bone-directed metastasis

**Figure 4:**
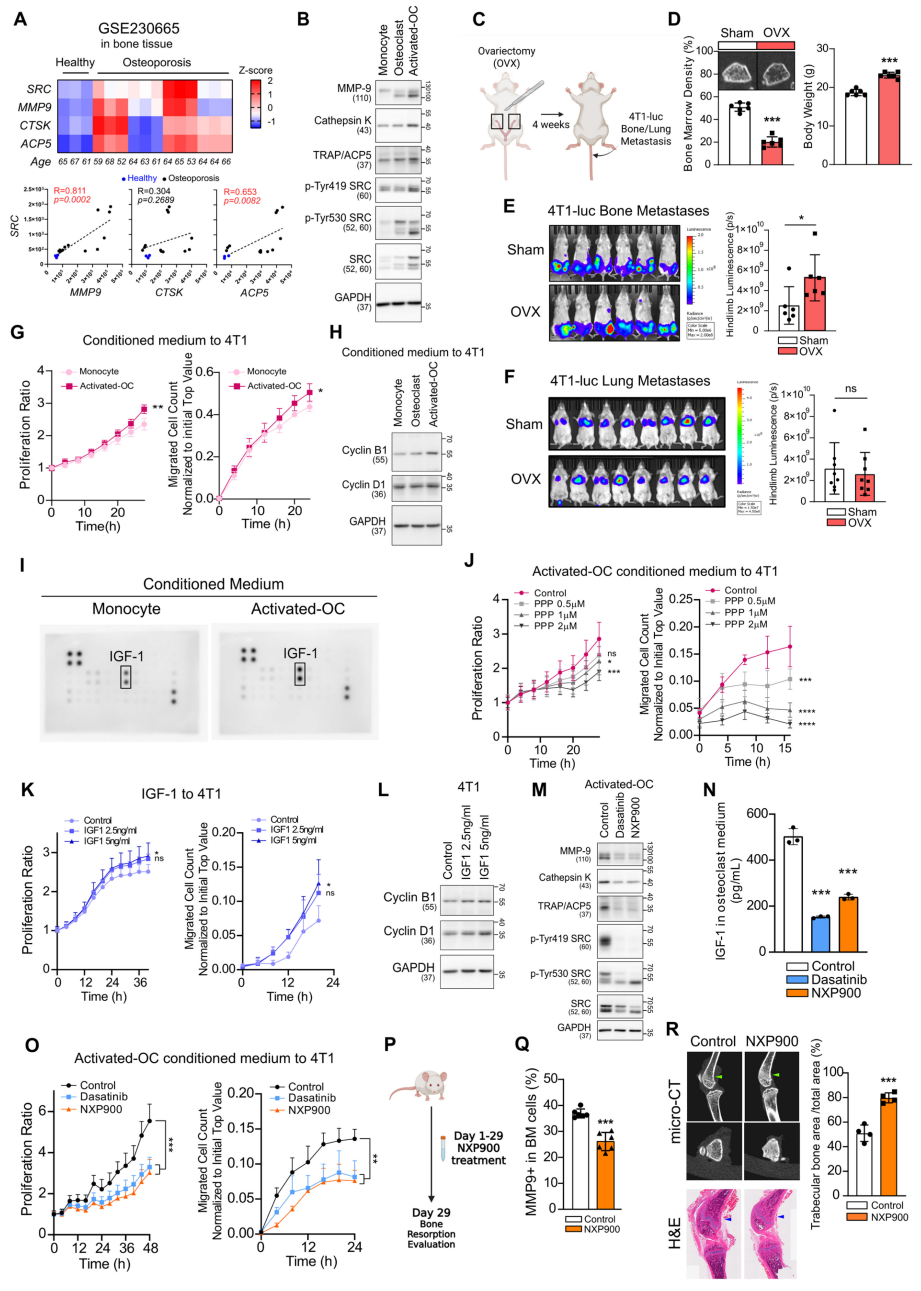
** SRC-driven osteoclast activation creates a bone microenvironment favorable for metastasis.** (A) Analysis of SRC and osteoclast activation markers in patients with osteoporosis using the NCBI public dataset GSE230665. (B) Evaluation of SRC and osteoclast activation markers during mouse primary osteoclast differentiation and activation. (C, D) Assessment of bone mineral density by micro-CT in ovariectomy (OVX)-induced osteoporosis model (4 weeks after surgery, N = 6/group). (E) Evaluation of bone metastasis induced by tail artery injection of 4T1-luc cells in OVX mice using IVIS Spectrum imaging at 1 week post-injection. N = 6/group. (F) Assessment of lung metastasis induced by tail vein injection under the same condition. N = 8/group. (G) Examination of migration and proliferation of mouse breast cancer cells treated with conditioned medium from monocytes or differentiated osteoclasts. (H) Analysis of proliferation marker expression in breast cancer cells following osteoclast-conditioned medium treatment. (I) Identification of growth factor differentially expressed in activated osteoclasts compared with monocytes. (J) Examination of migration and proliferation of mouse breast cancer cells treated with picropodophyllin (PPP)-containing conditioned medium from differentiated osteoclasts. (K, L) Evaluation of migration, proliferation, and proliferation marker expression in mouse breast cancer cells treated with recombinant IGF-1. (M) Assessment of osteoclast differentiation and activation after SRC inhibitor treatment. (N) Measurement of IGF-1 secretion from osteoclasts after SRC inhibition. (O) Evaluation of migration and proliferation of mouse breast cancer cells treated with conditioned medium from SRC-inhibited osteoclasts. SRC inhibitors were added during osteoclast differentiation. After replacing the medium with fresh medium, the medium was collected one day later and used to treat tumor cells. (P-R) Assessment of bone marrow MMP9⁺ cell frequency (%) by flow cytometry (N = 6/group, 2 weeks) and bone area fraction (trabecular bone area/total area) measured in the distal femur (as indicated by the arrows) by micro-CT (N = 4/group, 4 weeks) following daily oral administration of NXP900 (60 mg/kg). All data are presented as mean with standard deviation. Statistical significance was determined by two-tailed Student’s t-test or one-way ANOVA followed by Tukey’s test. *p < 0.05, **p < 0.01, ***p < 0.001, significant difference between indicated points.

Metastasis requires both cancer cell–intrinsic motility and the establishment of a permissive microenvironment at secondary sites 11-13, 19. Given that menopause-associated bone loss occurs in women over the age of 50 6, 7, 26, and time to diagnosis of bone metastasis is markedly reduced after this age (Figure 1C), menopause-related changes in bone density may create a microenvironment favorable for cancer cell colonization and promote the tropism of breast cancer toward bone. To investigate the potential role of SRC in pathological changes within the bone microenvironment, we analyzed samples from patients with osteoporosis (GSE230655). SRC expression was markedly elevated in these patients and positively correlated with osteoclast activation markers, including MMP9, CTSK, and ACP5 (Figure 4A). Furthermore, during differentiation and activation of primary mouse osteoclasts, SRC expression and its activity increased in parallel with osteoclast markers induction (Figure 4B).

Using organ-specific metastasis model in mice, we confirmed that intravenously injection of metastatic breast cancer cells predominantly induced lung metastasis, whereas intra-arterial injection preferentially induced bone (femur and tibia) metastasis (Figure S8A). In ovariectomized mice to mimic postmenopausal osteoporosis (Figure 4C-D), bone metastasis was significantly enhanced, whereas lung metastasis was not affected (Figure 4E-F). We further confirmed that the enhancement of bone metastasis was not attributable to sex-dependent differences (Figure S8B).

Next, we found that conditioned medium derived from activated osteoclasts increased proliferation, motility, and the expression of Cyclins in breast cancer cells (Figure 4G-H). Growth factor array analysis revealed a marked elevation of IGF-1 levels in this conditioned medium (Figure 4I), suggesting involvement of IGF-1 in primary effects on cancer cells. Inhibition of IGF-1 signaling using picropodophyllin (PPP), a selective inhibitor of IGF-1R, significantly reduced cancer cell proliferation and migration (Figure 4J). PPP effectively suppressed IGF-1–induced activation of the PI3K–AKT and MEK–ERK pathways in 4T1 cells, confirming that the observed effects are mediated through specific inhibition of the IGF-1 signaling axis (Figure S9). Conversely, recombinant mouse IGF-1 enhanced proliferation, migration, and the expression of Cyclins (Figure 4K-L). Treatment of activated-osteoclasts with SRC inhibitors significantly suppressed osteoclast activity (Figure 4M) and reduced IGF-1 secretion (Figure 4N). Conditioned medium collected from SRC inhibitor–treated osteoclasts reversed the osteoclast-mediated enhancement of breast cancer cell proliferation and motility (Figure 4O). Mechanistically, SRC inhibition in osteoclasts suppressed the NF-κB signaling pathway, which is required for osteoclast activation and IGF-1 synthesis (Figure S10) 48. Collectively, these findings indicate that menopause-associated osteoclast activation can transform bone into a tumor-promoting microenvironment, with IGF-1 serving as a key mediator.

Furthermore, oral administration of NXP900 reduced osteoclast activity and consequently increased bone density (Figure 4P-R). These results demonstrate that SRC-targeted therapy can prevent malignant remodeling of the bone microenvironment and thereby block extrinsic factors that drive breast cancer bone metastasis.

### A novel SRC-targeting inhibitor effectively suppresses breast cancer bone metastasis

**Figure 5:**
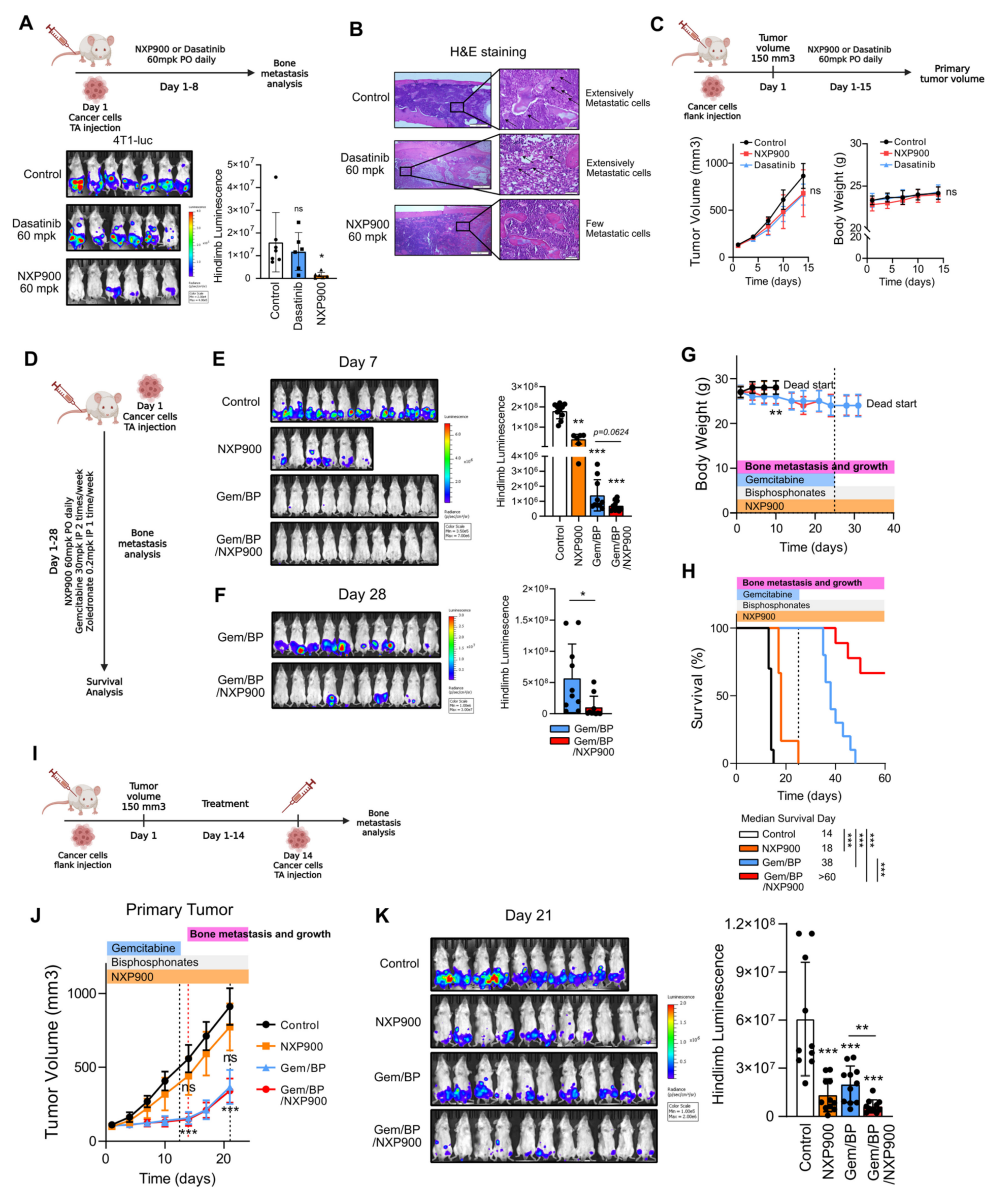
** NXP900 effectively suppresses breast cancer bone metastasis *in vivo*.** (A) Evaluation of the effect of SRC inhibitors on bone metastasis. NXP900 and dasatinib were orally administered daily at 60 mg/kg for 7 days. N = 6-7/group. (B) Assessment of bone metastatic lesions by H&E staining. (C) Evaluation of the effects of SRC inhibitors on primary tumor growth using the 4T1 flank subcutaneous model. NXP900 and dasatinib were orally administered daily at 60 mg/kg for 15 days. N = 5/group. (D) Evaluation of the therapeutic effects of NXP900 in combination with standard chemotherapy (gemcitabine/bisphosphonate; Gem/BP) on bone metastasis using the 4T1-luc murine breast cancer model. All treatments were initiated after induction of bone metastasis. NXP900 was given orally at 60 mg/kg daily, gemcitabine intraperitoneally at 30 mg/kg twice weekly, and zoledronate intraperitoneally at 0.2 mg/kg once weekly. Gemcitabine administration was discontinued on day 25. N = 6-10/group. (E) Quantification of bone metastasis on day 7 during ongoing gemcitabine treatment. (F) Quantification of bone metastasis on day 28 after gemcitabine withdrawal. (G) Assessment of body weight changes during treatment. (H) Survival analysis. (I) Evaluation of combination therapy in a subcutaneous 4T1 model. Mice bearing subcutaneous 4T1 tumors received Gem/BP chemotherapy with or without NXP900 to evaluate post-treatment bone metastasis. Gemcitabine was stopped on day 13, and bone metastasis was induced one day later. Drug doses were identical to those used in (D). N = 10-11/group. (J) Assessment of treatment effects on primary tumor growth. (K) Quantification of bone metastasis on day 21. All data are presented as mean with standard deviation. Statistical significance was determined by two-tailed Student’s t-test or one-way ANOVA followed by Tukey’s test. *p < 0.05, **p < 0.01, ***p < 0.001, significant difference between indicated points.

Next, we evaluated the efficacy of SRC-targeted therapies in a mouse model of breast cancer bone metastasis. Two SRC inhibitors were administered orally, followed by induction of breast cancer bone metastasis. Under these conditions, NXP900, but not dasatinib significantly reduced bone metastasis (Figure 5A-B), without affecting primary tumor growth and body weight gain (Figure 5C). Furthermore, NXP900 treatment exerted a comparable preventive effect on bone metastasis in an additional metastatic solid tumor model (B16F10 mouse melanoma, Figure S11). These results indicate that NXP900, a next-generation SRC inhibitor, exerts a robust suppressive effect on breast cancer bone metastasis.

Next, we evaluated the combined efficacy of NXP900 with the conventional regimen used for metastatic breast cancer, gemcitabine/bisphosphonate therapy (Gem/BP), in suppressing bone metastasis 49. Gemcitabine is a chemotherapeutic agent for metastatic breast cancer that directly inhibits cell proliferation and induces apoptosis 16. Bisphosphonates, widely used as a first-line therapy for osteoporosis, are also prescribed to alleviate bone-related complications in breast cancer patients 3, 9, 27. To mimic bone metastasis occurring during conventional regimen, bone metastasis was induced in mice undergoing Gem/BP treatment (Figure 5D). Owing to the potent cytotoxic effect of gemcitabine, co-administration of NXP900 did not show an additive benefit during the early treatment phase (Day 7) (Figure 5E). However, after gemcitabine withdrawal, which commonly occurs after several treatment cycles in clinical settings 50, metastatic tumor progression resumed, whereas combination treatment with NXP900 significantly suppressed bone metastasis progression and improved mouse survival (Figure 5F-H). We next assessed the preventive potential of NXP900 against bone metastasis occurring after completion of primary tumor therapy. For this, bone metastasis was induced following Gem/BP treatment of primary tumors (Figure 5I). In this setting, NXP900 co-administration did not further affect primary tumor control but exhibited an additional inhibitory effect on bone metastasis (Figure 5J-K). Together, these results indicate that SRC inhibition complements standard chemotherapy as a preventive strategy against breast cancer bone metastasis.

### SRC inhibition prevents bone metastasis by enhancing anti-tumor immunity in the pre-metastatic niche

**Figure 6:**
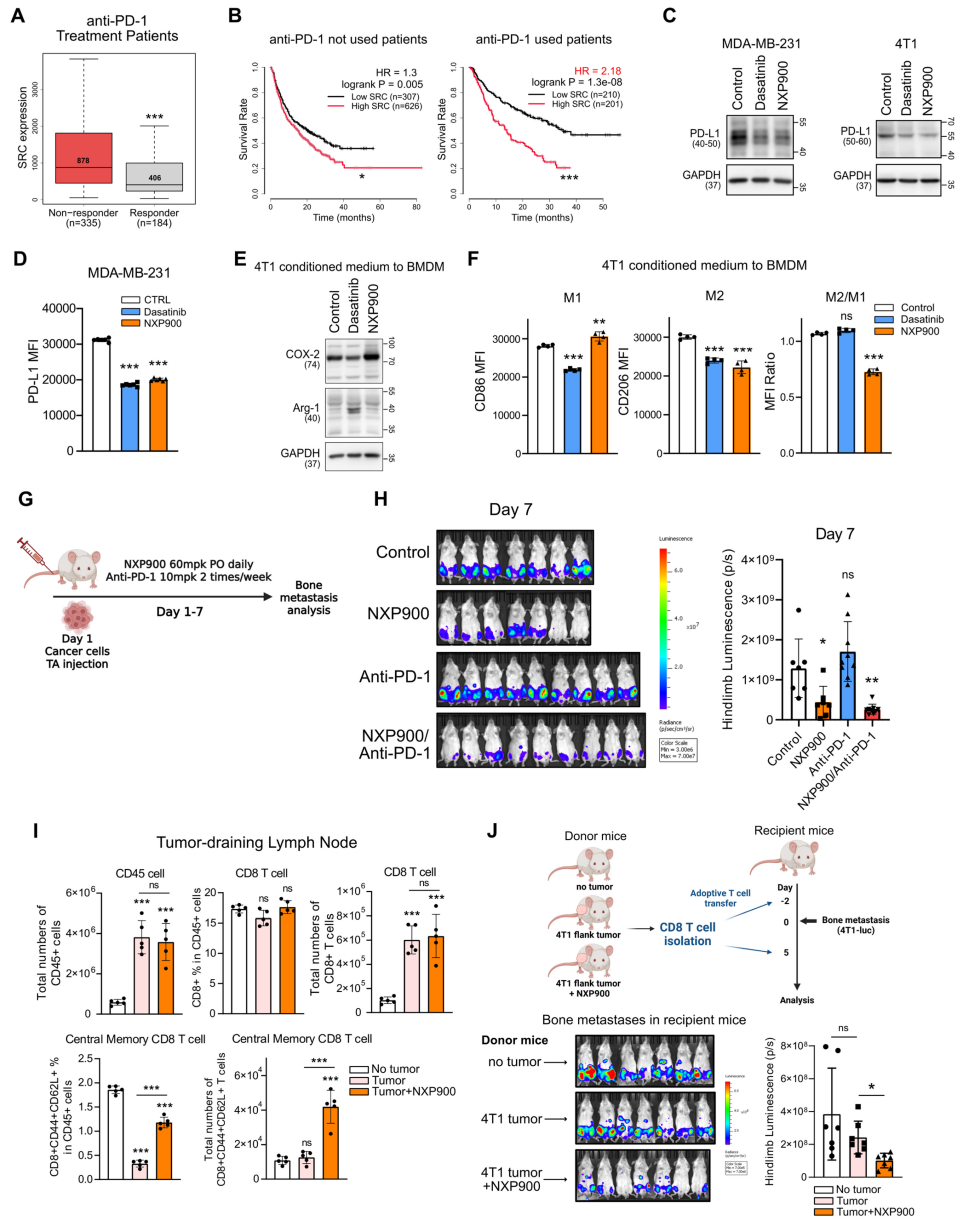
** SRC inhibition enhances anti-tumor immunity and prevents bone metastasis formation.** (A) SRC expression in patients responding or not responding to anti–PD-1 therapy. Analysis was performed using the ROC Plotter database. (B) Kaplan–Meier survival analysis of patients treated or not treated with anti–PD-1 therapy, stratified by SRC expression levels. Data were obtained from the Kaplan–Meier Plotter database. (C, D) PD-L1 expression in metastatic breast cancer cells following SRC inhibitor treatment analyzed by immunoblotting (C) and flow cytometry (D). (E, F) Conditioned medium from SRC inhibitor–treated 4T1 cancer cells was applied to bone marrow–derived macrophages to assess M1/M2 polarization by immunoblotting (E) and flow cytometry (F). (G, H) Evaluation of the combinatorial efficacy of anti–PD-1 antibody and NXP900 in a 4T1-luc bone metastasis model. Bone metastases were quantified one week after induction. NXP900 was administered orally at 60 mg/kg daily, and anti–PD-1 antibody was administered intraperitoneally at 10 mg/kg twice weekly. N = 7-9/group. (I) Quantification of CD8⁺ memory T cells following NXP900 treatment. 4T1-bearing mice received oral NXP900 (60 mg/kg daily) for one week. Tumor-draining (inguinal) lymph nodes were analyzed, and absolute immune cell numbers represent counts from a whole lymph node. N = 5/group. (J) Assessment of bone metastasis suppression by adoptive transfer of memory CD8⁺ T cells. CD8⁺ T cells were isolated from the spleen and inguinal lymph nodes of donor mice (as described in Fig. 6I) and intravenously transferred (2 × 10⁵ cells, twice) into recipient mice. Bone metastases were quantified one week after induction. N = 7/group. All data are presented as mean with standard deviation. Statistical significance was determined by two-tailed Student’s t-test or one-way ANOVA followed by Tukey’s test. *p < 0.05, **p < 0.01, ***p < 0.001, significant difference between indicated points.

In addition to stromal remodeling, immune evasion within the pre-metastatic niche also plays a critical role in enabling disseminated tumor cells to establish new secondary lesions 51. Therefore, blocking immune evasion in this niche is essential for the prevention and treatment of metastasis. Because we observed that SRC expression is associated with immune evasion pathways (Figure 1G), we next investigated whether SRC is involved in immune suppression during bone metastasis. Analysis of clinical datasets revealed that patients who failed to respond to anti-PD-1 therapy exhibited significantly higher SRC expression (Figure 6A), whereas anti-PD-1 therapy conferred an additional survival benefit in patients with low SRC expression (Figure 6B). These findings imply that SRC-mediated immune evasion in the pre-metastatic niche represents an important extrinsic factor that promotes bone metastasis and contributes to poor patient survival.

To assess the mechanistic contribution of SRC to tumor immune evasion, metastatic breast cancer cells were treated with SRC inhibitors. PD-L1 expression, a key mechanism of direct tumor immune evasion 52, was reduced by SRC inhibition (Figure 6C-D). In these cells, SRC inhibition suppressed the JNK–c-Jun signaling pathway, which is critical for PD-L1 expression (Figure S12) 53. Moreover, conditioned medium from SRC-inhibited breast cancer cells suppressed M2 macrophage polarization (Figure 6E-F), indicating attenuation of an indirect immune evasion mechanism 52. The murine 4T1 cell line is well known as a cold tumor with poor responsiveness to immune checkpoint blockade 54. Consistently, anti-PD-1 monotherapy did not directly prevent bone metastasis; however, combination therapy with NXP900 produced an additional preventive effect (Figure 6G-H).

Because cytotoxic chemotherapy can augment anti-tumor immunity by antigen release 55, we next considered whether enhanced immune memory following chemotherapy might contribute to metastasis prevention. Consistent with this notion, bone metastasis remained suppressed even after gemcitabine treatment was discontinued (Figure 5I-K), suggesting the presence of sustained anti-tumor immune response. We therefore examined whether SRC inhibition could promote immune memory formation. Although NXP900 administration did not directly eradicate primary tumors, it significantly increased the proportion of central memory CD8⁺ T cells in tumor-draining lymph nodes (Figure 6I and Figure S13A-B). Furthermore, adoptive transfer of these CD8⁺ T cells into recipient mice partially prevented bone metastasis even in the absence of additional drug treatment (Figure 6J and Figure S13C). Collectively, these findings indicate that SRC inhibition prevents metastasis by suppressing immune evasion and enhancing immune memory formation, thereby promoting anti-tumor immunity within the pre-metastatic niche.

## Discussion

Bone metastasis is a major complication in breast cancer, significantly reducing patient survival and quality of life 3, 4, 9. It commonly affects the ribs, spine, and long bones, leading to debilitating complications such as fractures, spinal cord compression, and chronic pain 4. Despite substantial advances in systemic therapies for breast cancer, effective strategies that specifically target bone metastasis remain limited. Current therapeutic strategies for metastatic breast cancer—including HER2-targeted therapies, CDK4/6 inhibitors, PARP inhibitors, and combination chemotherapy—have primarily target tumor-intrinsic mechanisms 16-18. While these approaches have improved outcomes in certain patient populations, their efficacy against bone metastasis remains insufficient, as disease progression is strongly influenced by the bone microenvironment. In particular, osteoclast activation and immune suppression are not adequately addressed, underscoring the need for therapeutic strategies that extend beyond tumor-intrinsic pathways.

SRC is one of the earliest identified oncogenes and has been investigated as a therapeutic target for more than five decades 56. Despite longstanding biological and pharmacological interest, SRC inhibition has not translated into meaningful clinical benefit in solid tumors. Emerging evidence indicates that SRC cannot be fully understood as a kinase-only target, as its biological activity is shaped not only by catalytic function but also by structural conformation, subcellular localization, and protein–protein interactions 33, 57-59. These complexities likely underlie the limited and inconsistent clinical efficacy of first-generation SRC inhibitors despite promising preclinical results.

Our study systematically demonstrates that SRC functions as a central regulator of breast cancer bone metastasis across multiple biological contexts. Analysis of clinical datasets revealed that SRC expression is elevated in metastatic breast cancer and is associated with poor patient prognosis (Figure 1A-F). Functionally, SRC promotes cancer cell motility through activation of the FAK–paxillin signaling axis and subsequent F-actin remodeling, which are suppressed by genetic or pharmacological inhibition of SRC (Figure 1H-N and Figure 2C-E). Mechanistically, distinct SRC activation states differentially regulate downstream signaling, with simultaneous inhibition of catalytic and scaffolding functions producing more complete suppression of FAK–paxillin–F-actin signaling and invadopodia formation compared with kinase inhibition alone (Figure 3B-E). In parallel, SRC facilitates bone-directed metastasis by promoting osteoclast activation and establishing a tumor-permissive bone microenvironment, partly through IGF-1–mediated signaling (Figure 4A-O). *In vivo*, SRC-targeted therapy suppresses osteoclast function, restores bone density, and significantly reduces bone tumor burden (Figure 4P-R and Figure 5A), with the next-generation SRC inhibitor NXP900 showing superior efficacy compared to dasatinib (Figure 5A-C). Furthermore, NXP900 in combination with conventional therapy provides durable suppression of metastatic progression and improves survival (Figure 5D-H). Beyond tumor-intrinsic motility and bone microenvironmental regulation, SRC also promotes immune evasion, as indicated by its association with anti-PD-1 resistance, regulation of PD-L1 expression, and induction of M2-like macrophage polarization (Figure 6A-F). Importantly, SRC inhibition enhances anti-tumor immunity and immune memory formation, leading to improved response to PD-1 blockade and sustained suppression of bone metastasis (Figure 6G-J).

A discrepancy between *in vitro* and *in vivo* efficacy was observed in our study. While no significant difference in migratory capacity was detected between SRC phospho-mutants *in vitro* (419DP/530DP vs 419DP/530P) (Figure 3B), inhibition of SRC scaffolding function (530P) consistently resulted in stronger suppression of downstream signaling, including reduced activation of FAK, paxillin, and F-actin remodeling (Figure 3D-E). A similar pattern was observed with pharmacological inhibition: although dasatinib and NXP900 showed comparable anti-migratory effects *in vitro* (Figure 1), only NXP900 demonstrated robust suppression of bone metastasis *in vivo* (Figure 5A). This discrepancy is unlikely to be attributed to differences in dosing or pharmacokinetics, suggesting a mechanistic basis. Notably, dasatinib was associated with compensatory FAK activation, whereas NXP900 more effectively suppressed the SRC–FAK–paxillin–F-actin signaling axis (Figure 2). These findings suggest that conventional *in vitro* migration assays may not fully capture F-actin–dependent invasive behaviors, such as invadopodia-mediated invasion. Although the precise mechanisms underlying this discrepancy remain to be fully defined, our data support the notion that next-generation SRC inhibitors targeting both catalytic and scaffolding functions may provide superior anti-metastatic efficacy *in vivo*.

These findings have important implications for how SRC should be interpreted therapeutically in solid tumors. Much of the prior translational effort has focused on kinase inhibition alone, particularly with dasatinib, yet clinical studies in metastatic breast cancer have shown limited efficacy, especially as monotherapy 60-62. Our results suggest that SRC should not be considered solely as a kinase-driven target, but rather as a multi-functional signaling regulator whose activity depends on both catalytic and scaffolding mechanisms. In this context, inhibitors capable of simultaneously targeting these functions may achieve more effective suppression of metastatic signaling pathways. This framework provides a potential explanation for the limited efficacy of first-generation SRC inhibitors and supports the development of next-generation agents with broader mechanistic activity.

These results also help reconcile conflicting findings in the SRC inhibitor literature. While many studies have reported anti-metastatic effects of SRC inhibition, others have shown increased metastatic potential following dasatinib treatment in the 4T1 metastasis model 63, 64. Such discrepancies likely reflect incomplete or context-dependent SRC inhibition, which allows compensatory activation of downstream signaling pathways. Our findings demonstrate that dual inhibition of catalytic and scaffolding functions produces more consistent suppression of metastatic signaling across cellular contexts. This suggests that the historical failure of SRC inhibitors in solid tumors may reflect limitations in targeting strategy rather than the biological irrelevance of SRC.

The bone microenvironment provides a critical context for SRC function. Bone metastases are clinically heterogeneous, presenting as osteolytic, osteoblastic, or mixed lesions 65. In breast cancer, osteolytic lesions predominate, making osteoclast-driven pathology particularly important 66. SRC is a well-established regulator of osteoclast activity, with a more prominent functional role in osteoclasts than in osteoblasts 67. Consistent with this, our data demonstrate that SRC promotes osteoclast activation and drives the formation of a tumor-permissive bone microenvironment. These findings highlight SRC as a key mediator of tumor–bone interactions and reinforce the concept that effective treatment of bone metastasis requires targeting both tumor cells and pathological bone remodeling. Nevertheless, because our study primarily focuses on osteoclast-driven mechanisms, the potential contributions of osteoblasts or other stromal components remain to be further investigated.

Our findings also suggest a translational strategy in which SRC inhibition is positioned as a mechanistically refined adjunct rather than a standalone therapy. NXP900 enhances the efficacy of conventional treatments and improves responsiveness to PD-1 blockade, indicating that its therapeutic benefit arises from simultaneous inhibition of tumor invasion, osteolytic remodeling, and immune evasion. Given that bone metastasis is sustained by complex interactions among tumor, stromal, and immune components, targeting a single compartment is unlikely to achieve durable control. However, the optimal combination strategies and treatment schedules for SRC-targeted therapies remain to be defined in clinical settings.

The interpretation of our *in vivo* findings should be considered in the context of the metastatic model used. Our study did not employ a spontaneous metastasis model. Instead, we used an intra-caudal arterial injection approach, which reliably induces bone metastasis and directly assesses tumor cell colonization and growth within the bone microenvironment. While this model provides strong mechanistic insight into metastatic outgrowth, it bypasses early steps of metastasis such as intravasation and dissemination. Similarly, the use of the 4T1 cell line in immunocompetent mice was intended to establish a robust and reproducible bone metastasis model rather than to represent a specific breast cancer subtype. Therefore, caution should be taken when extrapolating these findings to subtype-specific metastatic processes in human breast cancer.

Several limitations should be acknowledged. First, because our work was centered on bone colonization, it remains unclear whether SRC performs equally important or mechanistically distinct functions in metastasis to other organs such as lung, liver, or brain. Second, although inhibition of osteoclast-associated SRC activity is therapeutically attractive in predominantly osteolytic disease, long-term suppression of SRC-dependent bone remodeling could have unintended skeletal consequences and warrants dedicated safety evaluation. Third, while our data support a role for SRC in immune evasion, the downstream molecular circuitry connecting SRC inhibition to PD-L1 regulation and macrophage polarization remains incompletely resolved. Finally, the extent to which dependence on SRC varies among breast cancer subtypes remains unknown. Addressing these questions will be necessary for patient stratification and for determining whether next-generation SRC inhibitors should be advanced as monotherapy or as part of rational combination regimens.

## Conclusion

In summary, our study identifies SRC as a central signaling regulator of breast cancer bone metastasis by coordinating cancer cell–intrinsic motility (Figure 1-3), osteoclast-driven remodeling of the bone microenvironment (Figure 4), and immune evasion (Figure 6) through distinct functional mechanisms. Our work offers a mechanistic explanation for the historical limitations of SRC-targeted therapy in solid tumors. These findings support the continued development of scaffold-disrupting or conformation-selective SRC inhibitors as a more promising therapeutic strategy for breast cancer bone metastasis.

## Supplementary Material

Supplementary figures and tables.

Supplementary original blots.

## Data Availability

The data supporting the findings of this study are available from the corresponding author upon reasonable request.

## Abbreviations

Abl: Abelson tyrosine kinase
ACK: ammonium-chloride-potassium
ACP5: acid phosphatase 5
ACTG1: actin gamma 1
ANOVA: analysis of variance
BCR-ABL: breakpoint cluster region–Abelson
BP: bisphosphonate
CDK4/6: cyclin-dependent kinase 4/6
CTSK: cathepsin K
ELISA: enzyme-linked immunosorbent assay
ER: estrogen receptor
ERK: extracellular signal-regulated kinase
F-actin: filamentous actin
FAK: focal adhesion kinase
FBS: fetal bovine serum
G-actin: globular actin
H&E: hematoxylin and eosin
HEPES: 4-(2-hydroxyethyl)-1-piperazineethanesulfonic acid
HER2: human epidermal growth factor receptor 2
HRP: horseradish peroxidase
IDC: invasive ductal carcinoma
IDLC: invasive ductal and lobular carcinoma
IGF-1: insulin-like growth factor 1
IGF-1R: insulin-like growth factor 1 receptor
IHC: immunohistochemistry
IL-11: interleukin-11
ILC: invasive lobular carcinoma
JNK: c-Jun N-terminal kinase
M-CSF: macrophage colony-stimulating factor
MASLD: metabolic dysfunction-associated steatotic liver disease
MBC: Metastatic Breast Cancer
MEK: mitogen-activated protein kinase kinase
MMP9: matrix metalloproteinase 9
MPC: Metastatic Prostate Cancer
NF-κB: nuclear factor kappa B
OVX: ovariectomy
PARP: poly(ADP-ribose) polymerase
PBS: phosphate-buffered saline
PBST: phosphate-buffered saline containing 0.1% Tween 20
PD-1: programmed cell death protein 1
PD-L1: programmed death-ligand 1
PDGFRβ: platelet-derived growth factor receptor beta
PI3K: phosphoinositide 3-kinase
PPP: picropodophyllin
PR: progesterone receptor
PTK2: protein tyrosine kinase 2
PTHrP: parathyroid hormone-related protein
PXN: paxillin
RANKL: receptor activator of nuclear factor kappa-B ligand
RTKs: receptor tyrosine kinases
SDS-PAGE: sodium dodecyl sulfate–polyacrylamide gel electrophoresis
SFK: SRC family kinases
siRNA: small interfering RNA
TGF-β: transforming growth factor beta
TNBC: triple-negative breast cancer

## References

[1] Siegel RL, Kratzer TB, Giaquinto AN, Sung H, Jemal A (2025). Cancer statistics, 2025. CA Cancer J Clin.

[2] Harbeck N, Penault-Llorca F, Cortes J, Gnant M, Houssami N, Poortmans P (2019). Breast cancer. Nat Rev Dis Primers.

[3] Coleman RE, Croucher PI, Padhani AR, Clézardin P, Chow E, Fallon M (2020). Bone metastases. Nat Rev Dis Primers.

[4] Tsuzuki S, Park SH, Eber MR, Peters CM, Shiozawa Y (2016). Skeletal complications in cancer patients with bone metastases. Int J Urol.

[5] Łukasiewicz S, Czeczelewski M, Forma A, Baj J, Sitarz R, Stanisławek A (2021). Breast Cancer-Epidemiology, Risk Factors, Classification, Prognostic Markers, and Current Treatment Strategies-An Updated Review. Cancers (Basel).

[6] Wu YP, Chen WS, Xu SJ, Zhang N (2010). Osteoporosis as a potential contributor to the bone metastases. Med Hypotheses.

[7] Salamanna F, Borsari V, Contartese D, Nicoli Aldini N, Fini M (2018). Link between estrogen deficiency osteoporosis and susceptibility to bone metastases: A way towards precision medicine in cancer patients. Breast.

[8] Tsukamoto S, Kido A, Tanaka Y, Facchini G, Peta G, Rossi G (2021). Current Overview of Treatment for Metastatic Bone Disease. Curr Oncol.

[9] Ban J, Fock V, Aryee DNT, Kovar H (2021). Mechanisms, Diagnosis and Treatment of Bone Metastases. Cells.

[10] Yu Y, Ollodart J, Contino KF, Shiozawa Y (2023). Immunotherapy as a potential treatment approach for currently incurable bone metastasis. J Bone Miner Metab.

[11] Fares J, Fares MY, Khachfe HH, Salhab HA, Fares Y (2020). Molecular principles of metastasis: a hallmark of cancer revisited. Signal Transduct Target Ther.

[12] Lambert AW, Pattabiraman DR, Weinberg RA (2017). Emerging Biological Principles of Metastasis. Cell.

[13] Fidler IJ (2003). The pathogenesis of cancer metastasis: the 'seed and soil' hypothesis revisited. Nature Reviews Cancer.

[14] Choi YJ, Kim MJ, Lee YJ, Choi M, Shim WS, Park M (2025). Prevention of radiotherapy-induced pro-tumorigenic microenvironment by SFK inhibitors. Theranostics.

[15] Wang Z, Kim SY, Tu W, Kim J, Xu A, Yang YM (2023). Extracellular vesicles in fatty liver promote a metastatic tumor microenvironment. Cell Metab.

[16] Miglietta F, Bottosso M, Griguolo G, Dieci MV, Guarneri V (2022). Major advancements in metastatic breast cancer treatment: when expanding options means prolonging survival. ESMO Open.

[17] Yu J, Mu Q, Fung M, Xu X, Zhu L, Ho RJY (2022). Challenges and opportunities in metastatic breast cancer treatments: Nano-drug combinations delivered preferentially to metastatic cells may enhance therapeutic response. Pharmacol Ther.

[18] Al Sukhun S, Temin S, Barrios CH, Antone NZ, Guerra YC, Chavez-MacGregor M (2024). Systemic Treatment of Patients With Metastatic Breast Cancer: ASCO Resource-Stratified Guideline. JCO Glob Oncol.

[19] Esposito M, Guise T, Kang Y (2018). The Biology of Bone Metastasis. Cold Spring Harb Perspect Med.

[20] Kim H, Kim B, Il Kim S, Kim HJ, Ryu BY, Chung J (2019). S100A4 released from highly bone-metastatic breast cancer cells plays a critical role in osteolysis. Bone Research.

[21] Liang M, Ma Q, Ding N, Luo F, Bai Y, Kang F (2019). IL-11 is essential in promoting osteolysis in breast cancer bone metastasis via RANKL-independent activation of osteoclastogenesis. Cell Death & Disease.

[22] Zhang Y, Liang J, Liu P, Wang Q, Liu L, Zhao H (2022). The RANK/RANKL/OPG system and tumor bone metastasis: Potential mechanisms and therapeutic strategies. Front Endocrinol (Lausanne).

[23] Liu Y, Chen H, Chen T, Qiu G, Han Y (2024). The emerging role of osteoclasts in the treatment of bone metastases: rationale and recent clinical evidence. Front Oncol.

[24] Trivedi T, Pagnotti GM, Guise TA, Mohammad KS (2021). The Role of TGF-β in Bone Metastases. Biomolecules.

[25] Rieunier G, Wu X, Macaulay VM, Lee AV, Weyer-Czernilofsky U, Bogenrieder T (2019). Bad to the Bone: The Role of the Insulin-Like Growth Factor Axis in Osseous Metastasis. Clin Cancer Res.

[26] Eastell R, O'Neill TW, Hofbauer LC, Langdahl B, Reid IR, Gold DT (2016). Postmenopausal osteoporosis. Nature Reviews Disease Primers.

[27] Coleman R, Hadji P, Body JJ, Santini D, Chow E, Terpos E (2020). Bone health in cancer: ESMO Clinical Practice Guidelines. Ann Oncol.

[28] Ortiz MA, Mikhailova T, Li X, Porter BA, Bah A, Kotula L (2021). Src family kinases, adaptor proteins and the actin cytoskeleton in epithelial-to-mesenchymal transition. Cell Commun Signal.

[29] Li H, Zhao C, Tian Y, Lu J, Zhang G, Liang S (2020). Src family kinases and pulmonary fibrosis: A review. Biomed Pharmacother.

[30] Pelaz SG, Tabernero A (2022). Src: coordinating metabolism in cancer. Oncogene.

[31] Choi YJ, Choi M, Park J, Park M, Kim MJ, Lee JS (2024). Therapeutic strategy using novel RET/YES1 dual-target inhibitor in lung cancer. Biomed Pharmacother.

[32] Martellucci S, Clementi L, Sabetta S, Mattei V, Botta L, Angelucci A (2020). Src Family Kinases as Therapeutic Targets in Advanced Solid Tumors: What We Have Learned so Far. Cancers (Basel).

[33] Temps C, Lietha D, Webb ER, Li XF, Dawson JC, Muir M (2021). A Conformation Selective Mode of Inhibiting SRC Improves Drug Efficacy and Tolerability. Cancer Res.

[34] Dash S, Hanson S, King B, Nyswaner K, Foss K, Tesi N (2024). The SRC family kinase inhibitor NXP900 demonstrates potent antitumor activity in squamous cell carcinomas. J Biol Chem.

[35] Tang Z, Kang B, Li C, Chen T, Zhang Z (2019). GEPIA2: an enhanced web server for large-scale expression profiling and interactive analysis. Nucleic Acids Research.

[36] Menyhart O, Kothalawala WJ, Győrffy B (2025). A gene set enrichment analysis for cancer hallmarks. Journal of Pharmaceutical Analysis.

[37] Posta M, Győrffy B (2025). Pathway-level mutational signatures predict breast cancer outcomes and reveal therapeutic targets. Br J Pharmacol.

[38] Fekete JT, Győrffy B (2019). ROCplot.org: Validating predictive biomarkers of chemotherapy/hormonal therapy/anti-HER2 therapy using transcriptomic data of 3,104 breast cancer patients. Int J Cancer.

[39] Mathiesen RR, Fjelldal R, Liestøl K, Due EU, Geigl JB, Riethdorf S (2012). High-resolution analyses of copy number changes in disseminated tumor cells of patients with breast cancer. Int J Cancer.

[40] Xie L, Feng E, Li S, Chai H, Chen J, Li L (2023). Comparisons of gene expression between peripheral blood mononuclear cells and bone tissue in osteoporosis. Medicine (Baltimore).

[41] Jain E, Zañudo JGT, McGillicuddy M, Abravanel DL, Thomas BS, Kim D (2023). The Metastatic Breast Cancer Project: leveraging patient-partnered research to expand the clinical and genomic landscape of metastatic breast cancer and accelerate discoveries. medRxiv. 2023.

[42] Szklarczyk D, Nastou K, Koutrouli M, Kirsch R, Mehryary F, Hachilif R (2024). The STRING database in 2025: protein networks with directionality of regulation. Nucleic Acids Research.

[43] Guo Y, Arciero CA, Jiang R, Behera M, Peng L, Li X (2020). Different Breast Cancer Subtypes Show Different Metastatic Patterns: A Study from A Large Public Database. Asian Pac J Cancer Prev.

[44] Gao YM, Pei Y, Zhao FF, Wang L (2023). Osteoclasts in Osteosarcoma: Mechanisms, Interactions, and Therapeutic Prospects. Cancer Manag Res.

[45] Zhang S, Yu D (2012). Targeting Src family kinases in anti-cancer therapies: turning promise into triumph. Trends Pharmacol Sci.

[46] Kim LC, Song L, Haura EB (2009). Src kinases as therapeutic targets for cancer. Nature Reviews Clinical Oncology.

[47] Nelson LJ, Wright HJ, Dinh NB, Nguyen KD, Razorenova OV, Heinemann FS (2020). Src Kinase Is Biphosphorylated at Y416/Y527 and Activates the CUB-Domain Containing Protein 1/Protein Kinase C δ Pathway in a Subset of Triple-Negative Breast Cancers. Am J Pathol.

[48] Novack DV (2011). Role of NF-κB in the skeleton. Cell Research.

[49] El-Mabhouh AA, Nation PN, Abele JT, Riauka T, Postema E, McEwan AJ (2011). A conjugate of gemcitabine with bisphosphonate (Gem/BP) shows potential as a targeted bone-specific therapeutic agent in an animal model of human breast cancer bone metastases. Oncol Res.

[50] Crawford J, Herndon D, Gmitter K, Weiss J (2024). The impact of myelosuppression on quality of life of patients treated with chemotherapy. Future Oncol.

[51] Tufail M, Jiang C-H, Li N (2025). Immune evasion in cancer: mechanisms and cutting-edge therapeutic approaches. Signal Transduction and Targeted Therapy.

[52] Galassi C, Chan TA, Vitale I, Galluzzi L (2024). The hallmarks of cancer immune evasion. Cancer Cell.

[53] Ni Z, Sun P, Zheng J, Wu M, Yang C, Cheng M (2022). JNK Signaling Promotes Bladder Cancer Immune Escape by Regulating METTL3-Mediated m6A Modification of PD-L1 mRNA. Cancer Res.

[54] Lu Y, Houson HA, Gallegos CA, Mascioni A, Jia F, Aivazian A (2024). Evaluating the immunologically “cold” tumor microenvironment after treatment with immune checkpoint inhibitors utilizing PET imaging of CD4 + and CD8 + T cells in breast cancer mouse models. Breast Cancer Research.

[55] Opzoomer JW, Sosnowska D, Anstee JE, Spicer JF, Arnold JN (2019). Cytotoxic Chemotherapy as an Immune Stimulus: A Molecular Perspective on Turning Up the Immunological Heat on Cancer. Front Immunol.

[56] Simatou A, Simatos G, Goulielmaki M, Spandidos DA, Baliou S, Zoumpourlis V (2020). Historical retrospective of the SRC oncogene and new perspectives (Review). Mol Clin Oncol.

[57] Cui Y, Ali R, Clay M, Rossi P, Liu A, Yang D (2025). Conformational landscape adaptations enable processive phosphorylation by Src family kinases. Science.

[58] Beltran A, Naqvi MM, Faure AJ, Lehner B (2026). The allosteric landscape of the Src kinase. Sci Adv.

[59] Delaveris CS, Loudermilk RP, Pandey A, Remesh SG, Peters-Clarke TM, Ganjave SD (2026). Autophagolysosomal exocytosis inverts Src kinase onto the cell surface in cancer. Science.

[60] Schott AF, Barlow WE, Van Poznak CH, Hayes DF, Moinpour CM, Lew DL (2016). Phase II studies of two different schedules of dasatinib in bone metastasis predominant metastatic breast cancer: SWOG S0622. Breast Cancer Research and Treatment.

[61] Herold CI, Chadaram V, Peterson BL, Marcom PK, Hopkins J, Kimmick GG (2011). Phase II trial of dasatinib in patients with metastatic breast cancer using real-time pharmacodynamic tissue biomarkers of Src inhibition to escalate dosing. Clin Cancer Res.

[62] Chapdelaine AG, Sun G (2025). Molecular Pharmacology of Dasatinib Provides Unique Insights into the Mechanistic Basis of Success and Failure of Targeted Cancer Therapy. ACS Pharmacol Transl Sci.

[63] Hughes VS, Siemann DW (2018). Treatment with Src inhibitor Dasatinib results in elevated metastatic potential in the 4T1 murine mammary carcinoma model. Tumor Microenviron.

[64] Kennedy LC, Gadi V (2018). Dasatinib in breast cancer: Src-ing for response in all the wrong kinases. Ann Transl Med.

[65] Wang M, Xia F, Wei Y, Wei X (2020). Molecular mechanisms and clinical management of cancer bone metastasis. Bone Res.

[66] Venetis K, Piciotti R, Sajjadi E, Invernizzi M, Morganti S, Criscitiello C (2021). Breast Cancer with Bone Metastasis: Molecular Insights and Clinical Management. Cells.

[67] Matsubara T, Yasuda K, Mizuta K, Kawaue H, Kokabu S (2022). Tyrosine Kinase Src Is a Regulatory Factor of Bone Homeostasis. Int J Mol Sci.

