# Supplementary material for: Inhibition of SRC prevents bone metastasis of breast cancer by blocking metastatic cell motility and bone directionality
Source: Theranostics. 2026 May 18;16(12):6928–47. doi: 10.7150/thno.130647 (PMC13232475; doi:10.7150/thno.130647)
Supplement: Supplementary file 1 — Supplementary figures and tables. [file thnov16p6928s1.pdf]

Supplementary Figures and Tables

Inhibition of SRC prevents bone metastasis of breast cancer by blocking metastatic cell motility and bone directionality

Yong June Choi[1,2,†], Minju Kwon[1,†], Myung Jun Kim[1], Munkyung Choi[1], Phuong Thao Tran[1], Yujeong Lee[1], Wan Seob Shim[1], Minjae Kang[1], Seungseok Oh[1], Sung-Chul Lim[3], Yong-Chul Kim[4], Keon Wook Kang[1,*]

[†]These authors contributed equally.

[*]Corresponding Author, E-mail: kwkang@snu.ac.kr

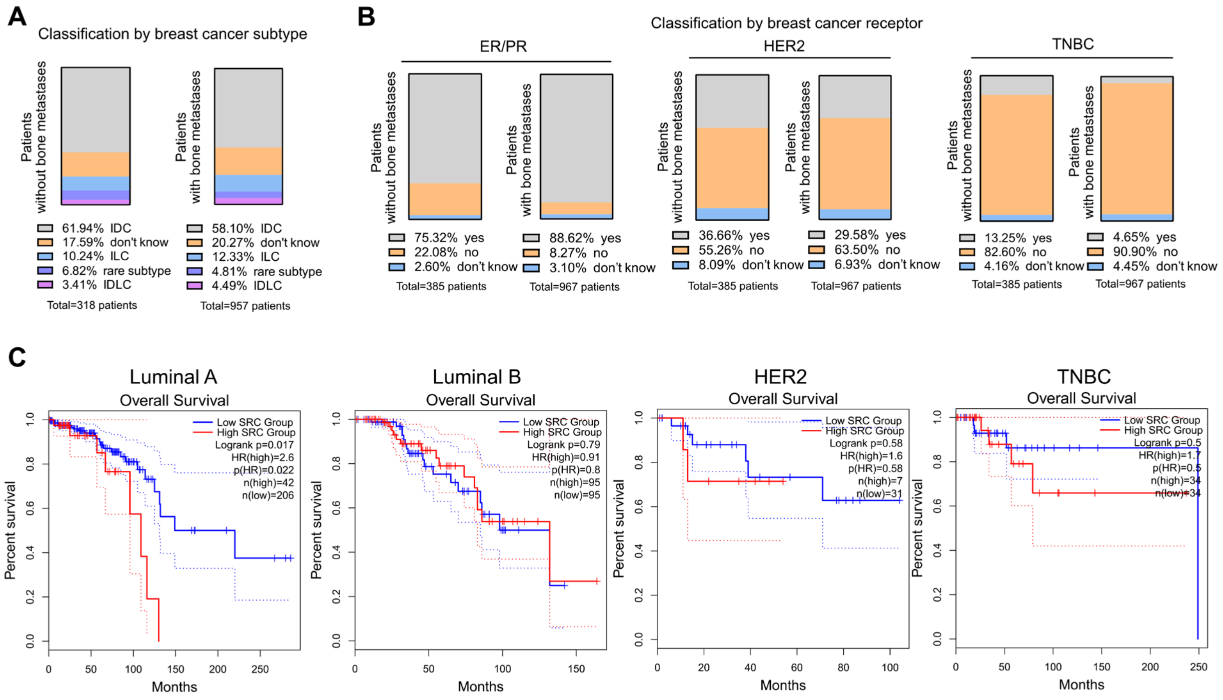

Figure S1, related to Figure 1. Distribution of breast cancer subtypes and receptor profiles in bone metastatic cohorts.

(A, B) Distribution of breast cancer subtypes in patients with and without bone metastasis. (The MBC Project, 2015–2025). (C) Survival analysis of breast cancer patients stratified by SRC expression across different subtypes. Data were obtained from the GEPIA public database. Abbreviations: IDC, invasive ductal carcinoma; ILC, invasive lobular carcinoma; IDLC, invasive ductal and lobular carcinoma.

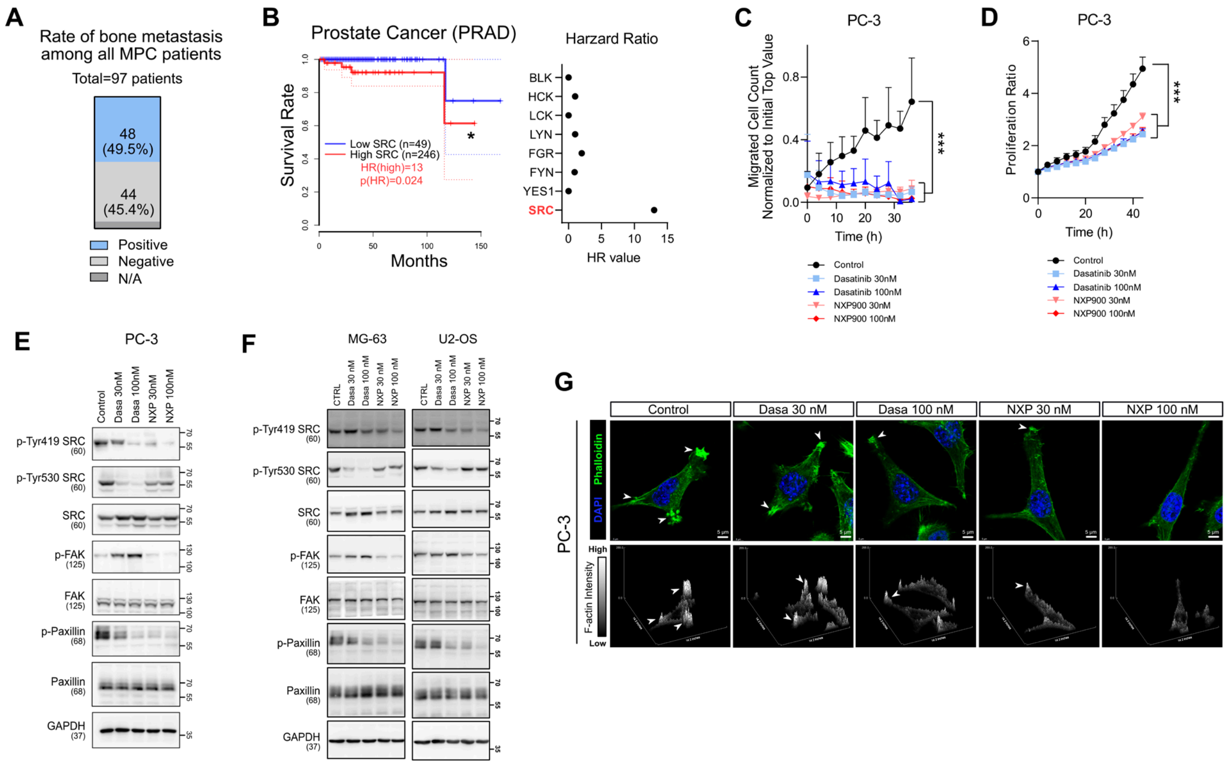

Figure S2, related to Figure 1-2. SRC contributes to cell motility and poor prognosis in prostate cancer.

(A) Bone metastasis cases in metastatic prostate cancer patients analyzed through prospective and retrospective investigations [The Metastatic Prostate Cancer (MPC) project, http://mpcproject.org/]. (B) Survival rate of prostate cancer patients according to SRC expression (left) and hazard ratios of SRC family kinases (right). Data were obtained from the GEPIA public database. (C, D) Effects of SRC inhibitors on transwell migration and proliferation of metastatic prostate cancer cells. (E) Changes in SRC, FAK, and paxillin activation in prostate cancer cells following treatment with SRC inhibitors. (F) Changes in SRC, FAK, and paxillin activation in osteosarcoma cells treated with SRC inhibitors. (G) Representative confocal images of prostate cancer cells treated with vehicle or SRC inhibitors, showing F-actin–rich invadopodia structures. Cells were stained with phalloidin (green) to visualize F-actin and DAPI (blue) to label nuclei. Arrowheads indicate invadopodia-like actin puncta. Z-stack reconstruction with orthogonal projections and surface plots is shown to visualize the spatial organization, vertical distribution, and relative intensity of F-actin

structures in control and SRC inhibitor–treated cells. All data were presented as mean with SD. Statistical significance of the differences was determined by one-way ANOVA followed by Tukey's test. *$p < 0.05$, **$p < 0.01$, ***$p < 0.001$, significant difference between indicated points.

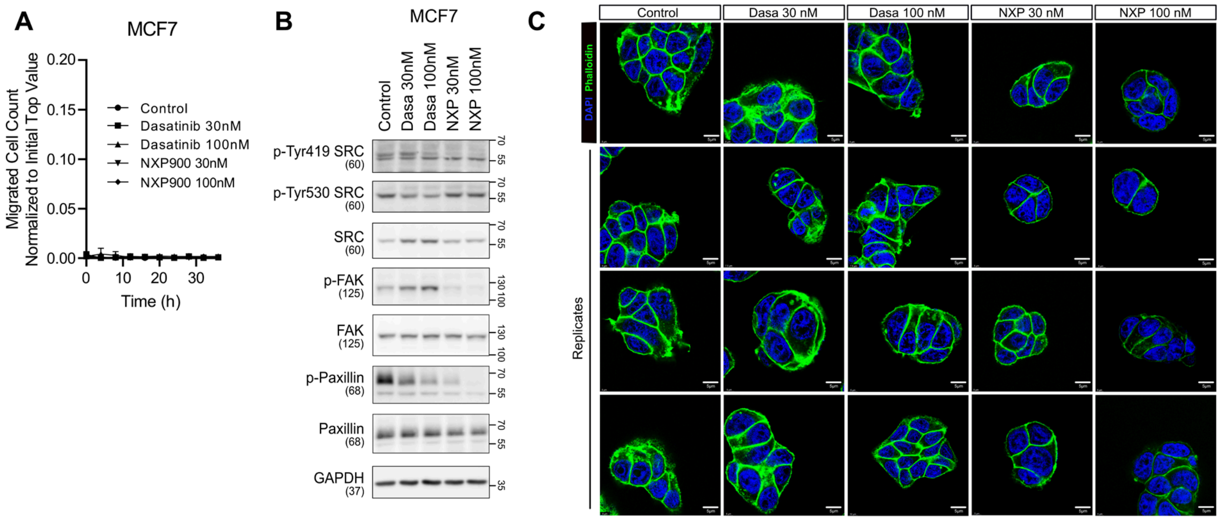

Figure S3, related to Figure 1-2. SRC inhibition suppresses SRC–paxillin–F-actin signaling in ER-positive breast cancer.

(A) Effects of SRC inhibitors on transwell migration of ER-positive human breast cancer cells. (B) Changes in SRC, FAK, and paxillin activation in ER-positive human breast cancer cells following treatment with SRC inhibitors. (C) Representative confocal images of ER-positive human breast cancer cells treated with vehicle or SRC inhibitors, showing F-actin. Cells were stained with phalloidin (green) to visualize F-actin and DAPI (blue) to label nuclei.

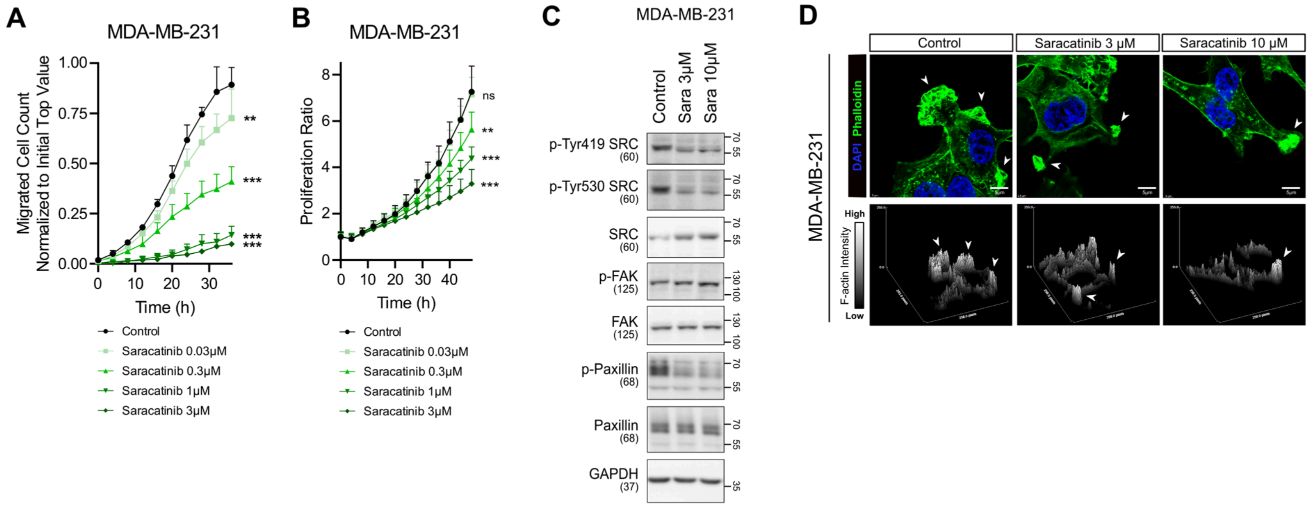

Figure S4, related to Figure 1-2. Validation of SRC-dependent signaling using a conventional SRC inhibitor, Saracatinib.

(A, B) Effects of Saracatinib on transwell migration and proliferation of metastatic breast cancer cells. (C) Changes in SRC, FAK, and paxillin activation in metastatic breast cancer cells following treatment with Saracatinib. (D) Representative confocal images of prostate cancer cells treated with vehicle or Saracatinib, showing F-actin–rich invadopodia structures. Cells were stained with phalloidin (green) to visualize F-actin and DAPI (blue) to label nuclei. Arrowheads indicate invadopodia-like actin puncta. Z-stack reconstruction with orthogonal projections and surface plots is shown to visualize the spatial organization, vertical distribution, and relative intensity of F-actin structures in control and SRC inhibitor–treated cells. All data were presented as mean with SD. Statistical significance of the differences was determined by one-way ANOVA followed by Tukey's test. $*p < 0.05$, $**p < 0.01$, $***p < 0.001$, significant difference between indicated points.

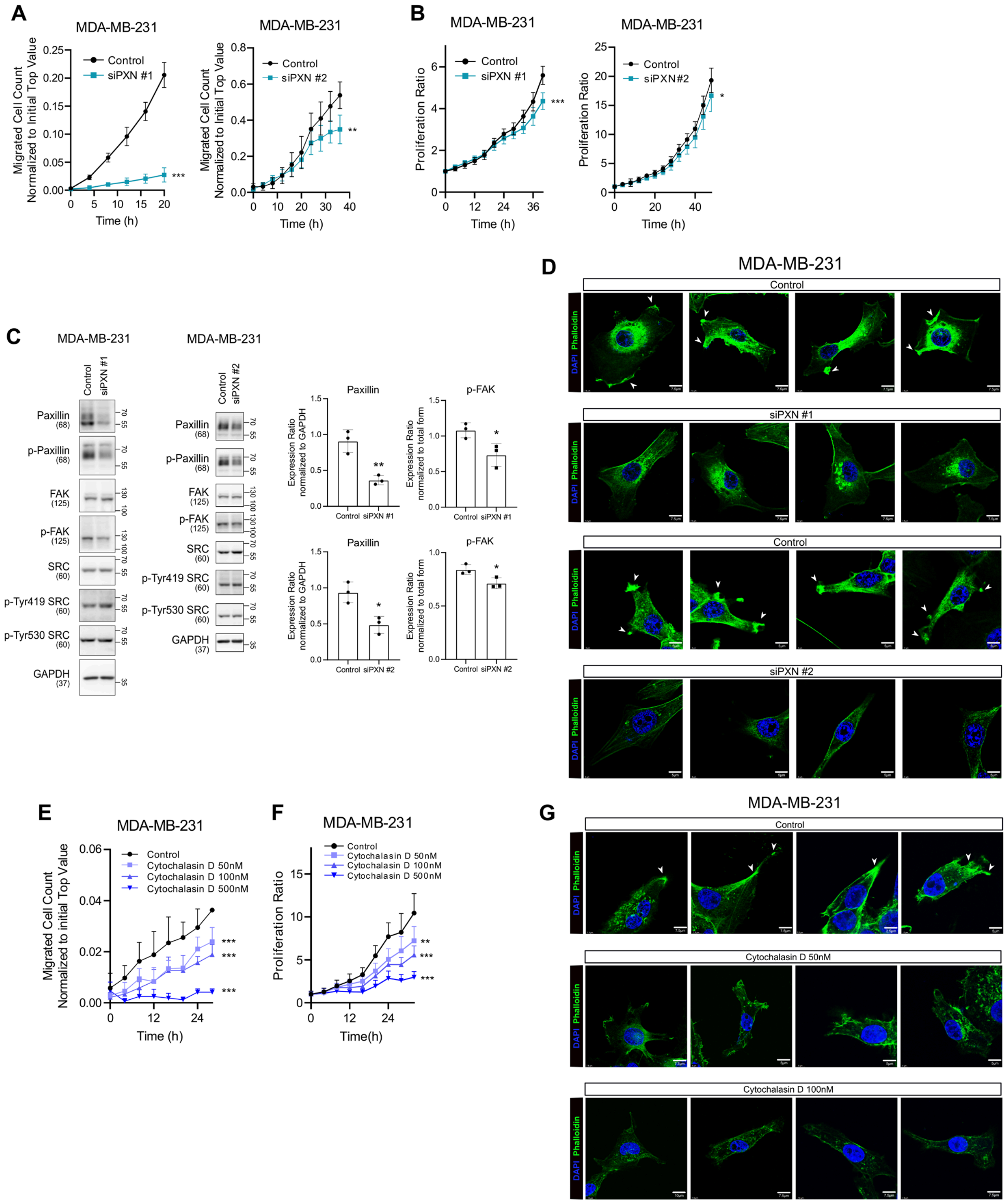
A
MDA-MB-231
Migrated Cell Count Normalized to Initial Top Value
Control
siPXN #1
siPXN #2
Time (h)
***
**
B
MDA-MB-231
Proliferation Ratio
Control
siPXN #1
siPXN#2
Time (h)
***
*
C
MDA-MB-231
Control
siPXN #1
siPXN #2
Paxillin (68)
p-Paxillin (68)
FAK (125)
p-FAK (125)
SRC (60)
p-Tyr419 SRC (60)
p-Tyr530 SRC (60)
GAPDH (37)
Paxillin
p-FAK
Expression Ratio normalized to GAPDH
Expression Ratio normalized to total form
D
MDA-MB-231
Control
siPXN #1
siPXN #2
DAPI Phalloidin
E
MDA-MB-231
Migrated Cell Count Normalized to initial Top Value
Control
Cytochalasin D 50nM
Cytochalasin D 100nM
Cytochalasin D 500nM
Time (h)
F
MDA-MB-231
Proliferation Ratio
Control
Cytochalasin D 50nM
Cytochalasin D 100nM
Cytochalasin D 500nM
Time(h)
G
MDA-MB-231
Control
Cytochalasin D 50nM
Cytochalasin D 100nM
DAPI Phalloidin

Figure S5, related to Figure 2. Validation of the link between paxillin-mediated actin polymerization and cell motility.

(A, B) Changes in transwell migration and proliferation of breast cancer cells following paxillin knockdown. (C) Alterations in the activation of paxillin, FAK, and SRC after paxillin knockdown. (D) Representative confocal images of breast cancer cells following paxillin knockdown, showing F-actin–rich invadopodia structures. (E, F) Effects of cytochalasin D on transwell migration and proliferation of breast cancer cells. (G) Representative confocal images of breast cancer cells treated with vehicle or SRC inhibitors, showing F-actin–rich invadopodia structures. Cells were stained with phalloidin (green) to visualize F-actin and DAPI (blue) to label nuclei. Arrowheads indicate invadopodia-like actin puncta. All data were presented as mean with SD. Statistical significance of the differences was determined by two-tailed Student's t-test or one-way ANOVA followed by Tukey's test. *$p < 0.05$, **$p < 0.01$, ***$p < 0.001$, significant difference between indicated points. Detailed information on individual siRNAs is provided in the Methods section.

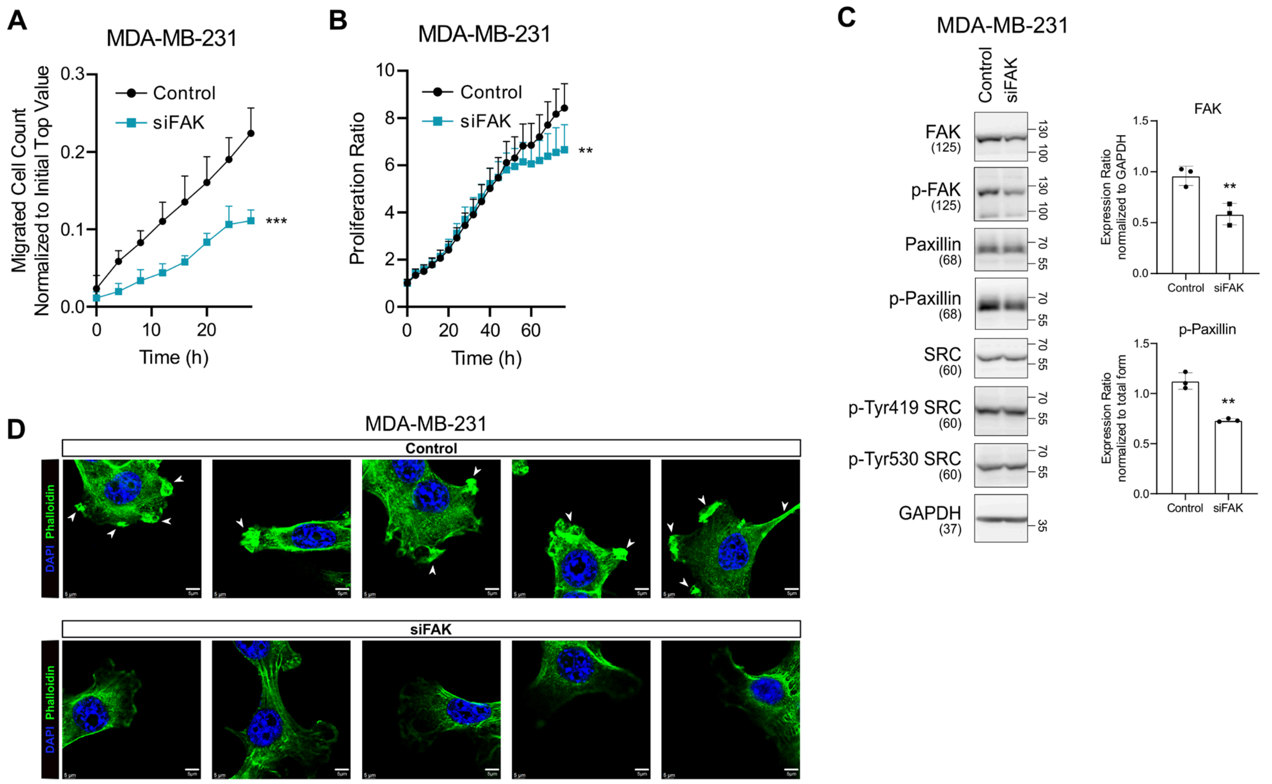

Figure S6, related to Figure 2. Role of FAK in SRC-mediated regulation of cell motility.

(A, B) Changes in transwell migration and proliferation of breast cancer cells following FAK knockdown. (C) Alterations in the activation of paxillin, FAK, and SRC after FAK knockdown. (D) Representative confocal images of breast cancer cells following FAK knockdown, showing F-actin–rich invadopodia structures. All data were presented as mean with SD. Statistical significance of the differences was determined by two-tailed Student's t-test. $*p < 0.05$, $**p < 0.01$, $***p < 0.001$, significant difference between indicated points. Detailed information on individual siRNAs is provided in the Methods section.

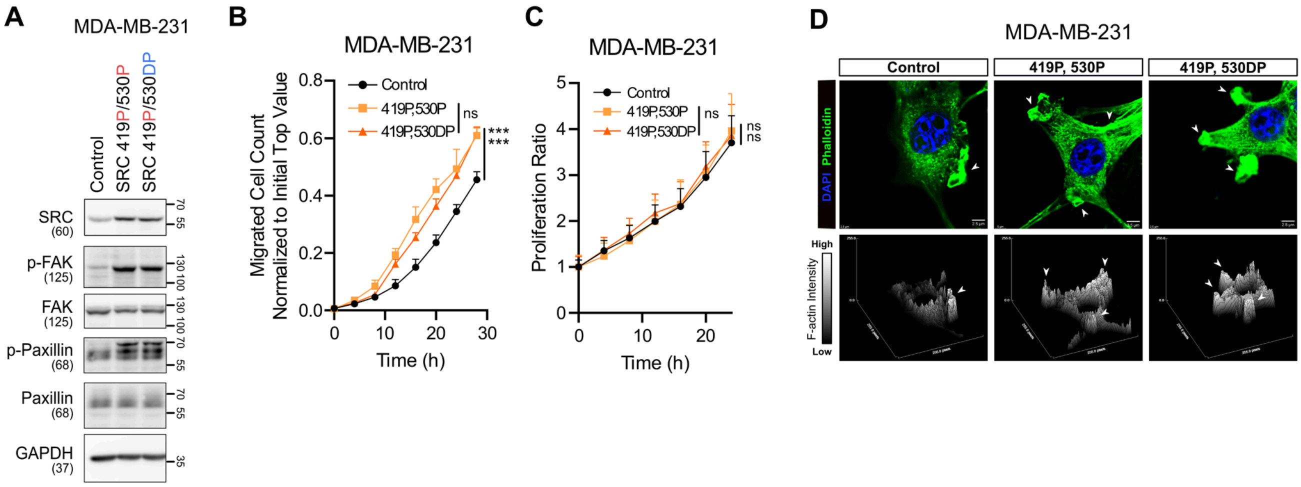

Figure S7, related to Figure 3. Validation of phosphorylation-dependent SRC signaling.

(A) Changes in FAK, and paxillin activation following phospho-mutant SRC plasmids overexpression. (B, C) Changes in transwell migration and proliferation of breast cancer cells following phospho-mutant SRC plasmids overexpression. (D) Representative confocal images of MDA-MB-231 breast cancer cells expressing SRC phospho-mutant constructs (419P/530P, 419P/530DP), showing F-actin–rich invadopodia-like structures. Cells were stained with phalloidin (green) to visualize F-actin and DAPI (blue) to label nuclei. Arrowheads indicate invadopodia-like actin puncta. Z-stack images were reconstructed and displayed as surface plots to visualize the spatial organization, vertical distribution, and relative intensity of F-actin signals across the indicated SRC phospho-mutant conditions. All data were presented as mean with SD. Statistical significance of the differences was determined by one-way ANOVA followed by Tukey's test. *$p < 0.05$, **$p < 0.01$, ***$p < 0.001$, significant difference between indicated points.

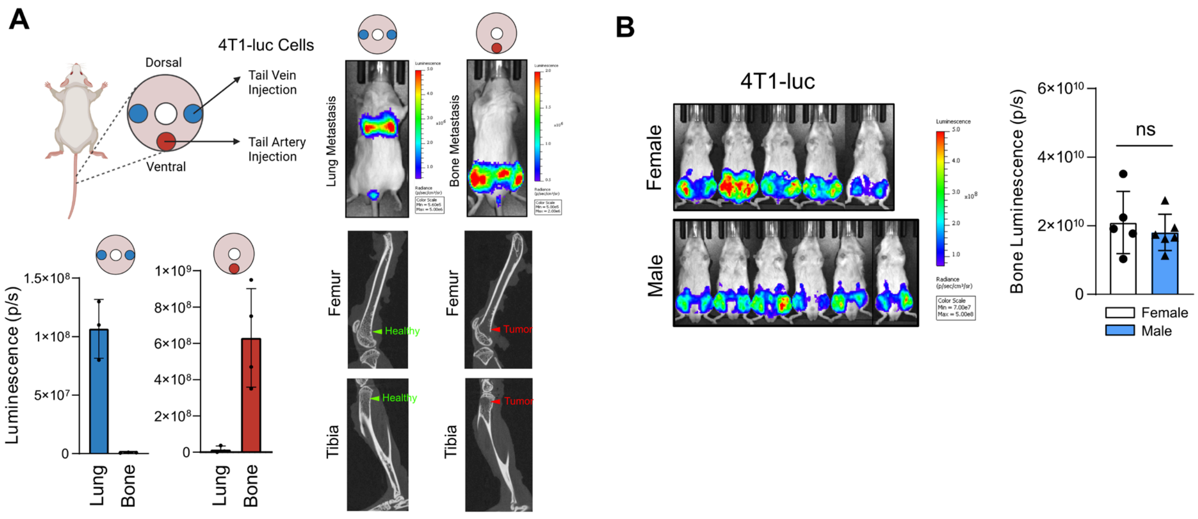

Figure S8, related to Figure 4. Organ-specific metastasis models and sex-dependent effects.

(A) Establishment of bone and lung metastasis models using murine metastatic breast cancer cells. Quantitative assessment was performed using IVIS Spectrum imaging, and qualitative bone evaluation was conducted by micro-CT. (B) Evaluation of sex-dependent differences in breast cancer bone metastasis. Female and male mice were injected with 4T1-luc cells, and metastatic burden was quantified 1 week after injection. N = 5-6/group. All data were presented as mean with SD. Statistical significance of the differences was determined by two-tailed Student's t-test. *$p < 0.05$, **$p < 0.01$, ***$p < 0.001$, significant difference between indicated points.

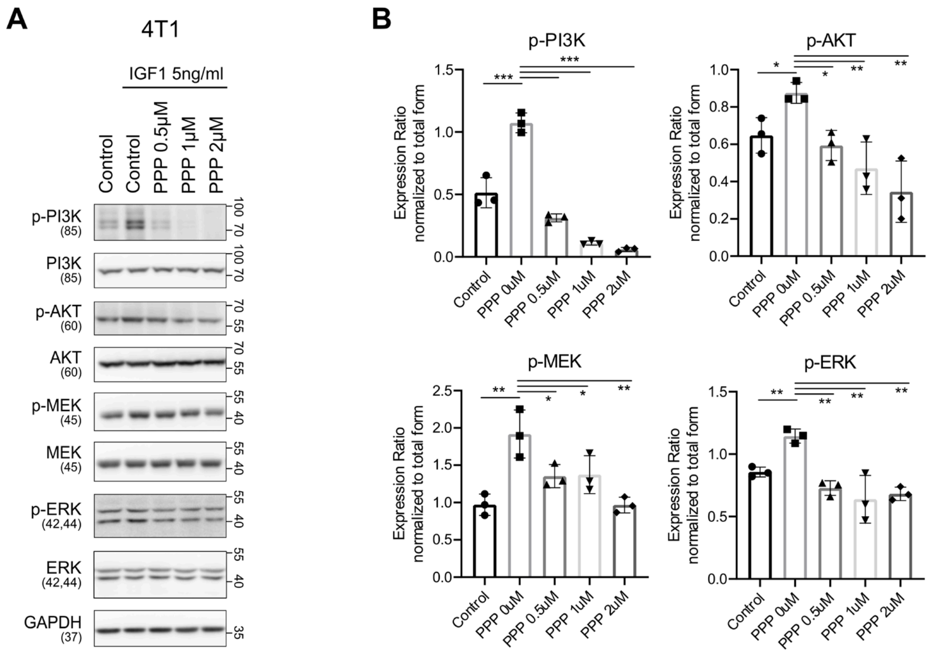

Figure S9, related to Figure 4. Inhibition of IGF-induced tumor growth signaling by picropodophyllin (PPP).

(A) Assessment of IGF-dependent PI3K–AKT and RAS signaling in breast cancer cells following picropodophyllin (PPP) treatment. (B) Densitometric quantification of Western blot results. All data were presented as mean with SD. Statistical significance of the differences was determined by two-tailed Student's t-test or one-way ANOVA followed by Tukey's test. *p < 0.05, **p < 0.01, ***p < 0.001, significant difference between indicated points.

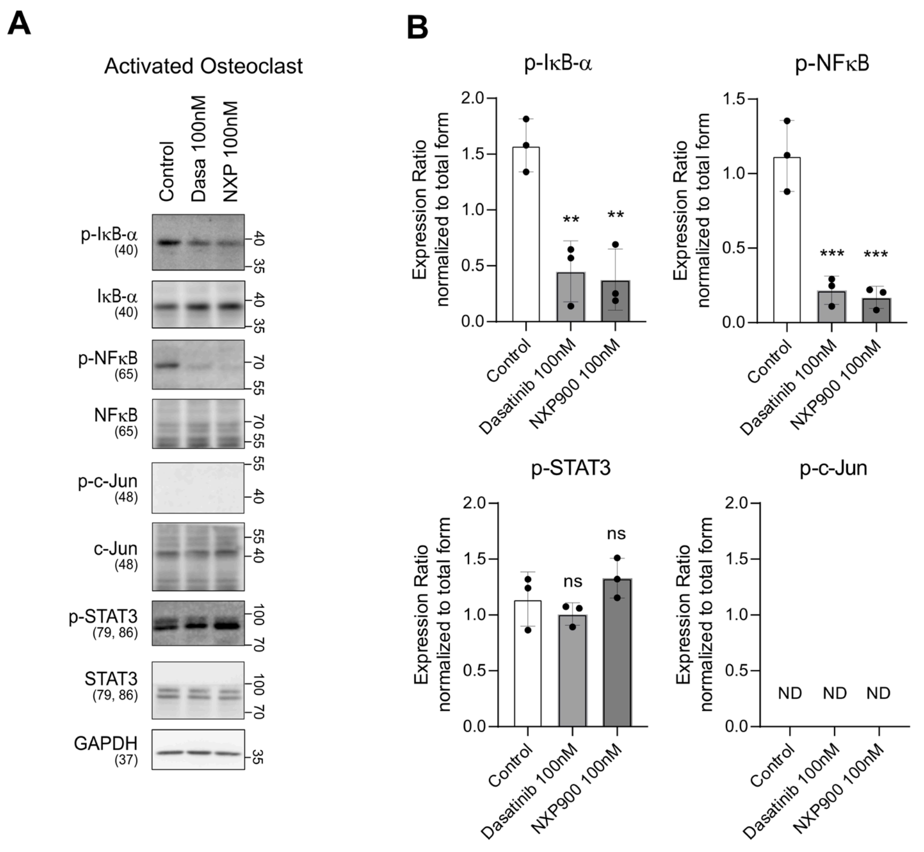

Figure S10, related to Figure 4. SRC inhibition modulates transcription factors associated with osteoclast activation.

(A) Assessment of STAT3, NF-κB, and c-Jun expression in mouse primary osteoclasts following SRC inhibitors treatment. (B) Densitometric quantification of Western blot results. All data were presented as mean with SD. Statistical significance of the differences was determined by one-way ANOVA followed by Tukey's test. $*p < 0.05$, $**p < 0.01$, $***p < 0.001$, significant difference between indicated points.

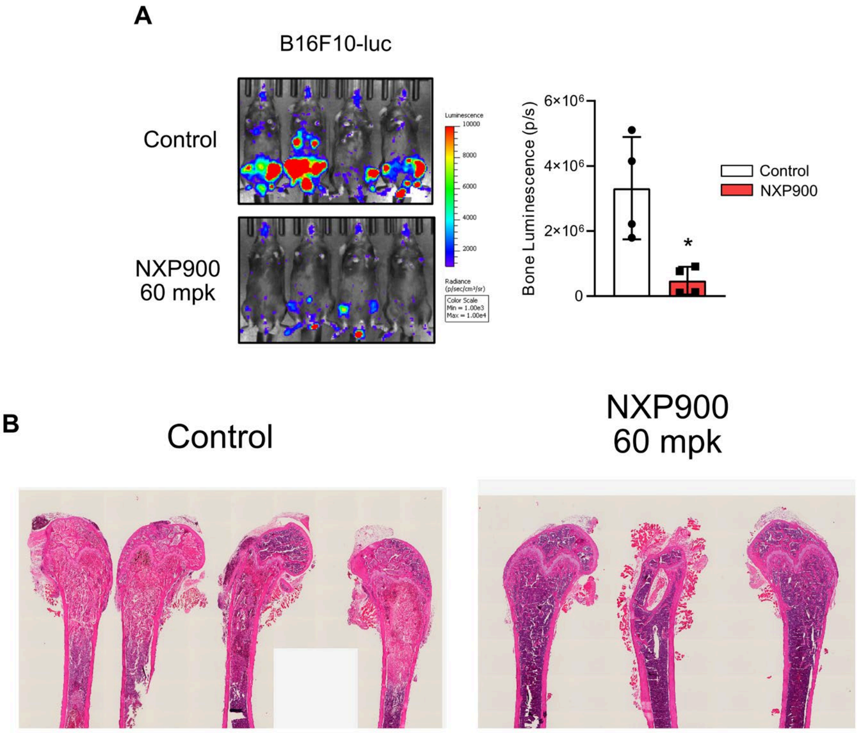

Figure S11, related to Figure 5. Efficacy of NXP900 across different metastatic solid tumor model.

(A) Evaluation of the effect of NXP900 on bone metastasis using the B16F10-luc murine metastatic melanoma cancer model. NXP900 was orally administered daily at 60 mg/kg for 7 days. N = 4/group. (B) Assessment of bone metastatic lesions by H&E staining. All data were presented as mean with SD. Statistical significance of the differences was determined by two-tailed Student's t-test. *$p < 0.05$, **$p < 0.01$, ***$p < 0.001$, significant difference between indicated points.

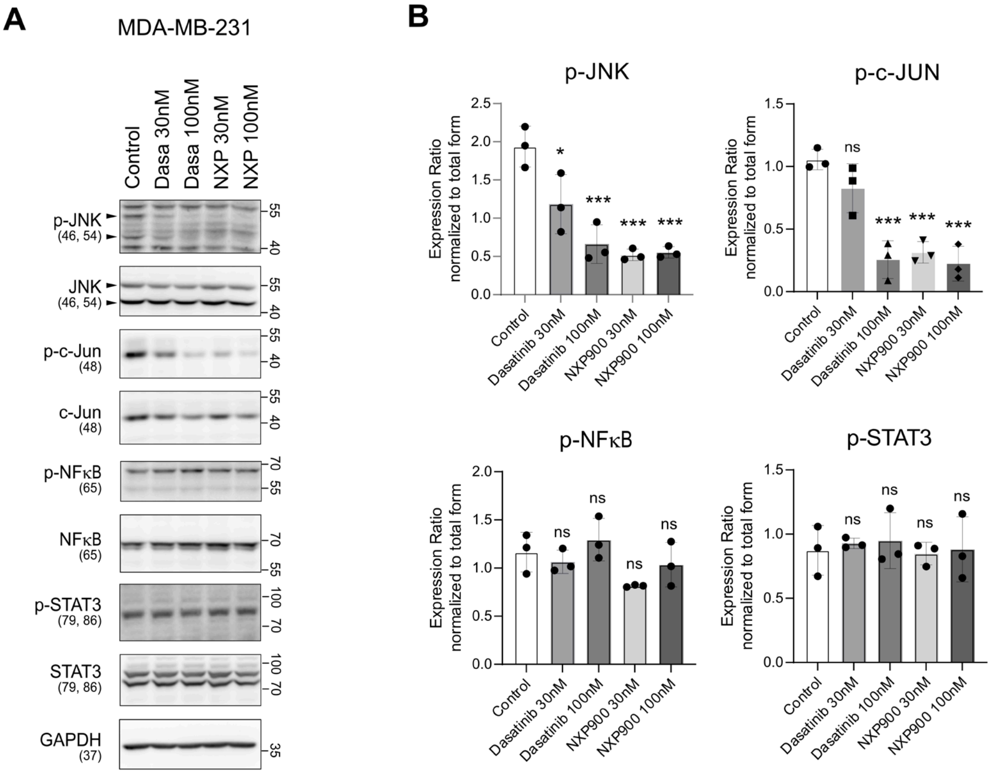

Figure S12, related to Figure 6. SRC inhibition regulates transcription factors associated with PD-L1 expression.

(A) Assessment of transcription factor level regulating PD-L1 in breast cancer cells following SRC inhibitors treatment. (B) Densitometric quantification of Western blot results. All data were presented as mean with SD. Statistical significance of the differences was determined by one-way ANOVA followed by Tukey's test. $*p < 0.05$, $**p < 0.01$, $***p < 0.001$, significant difference between indicated points.

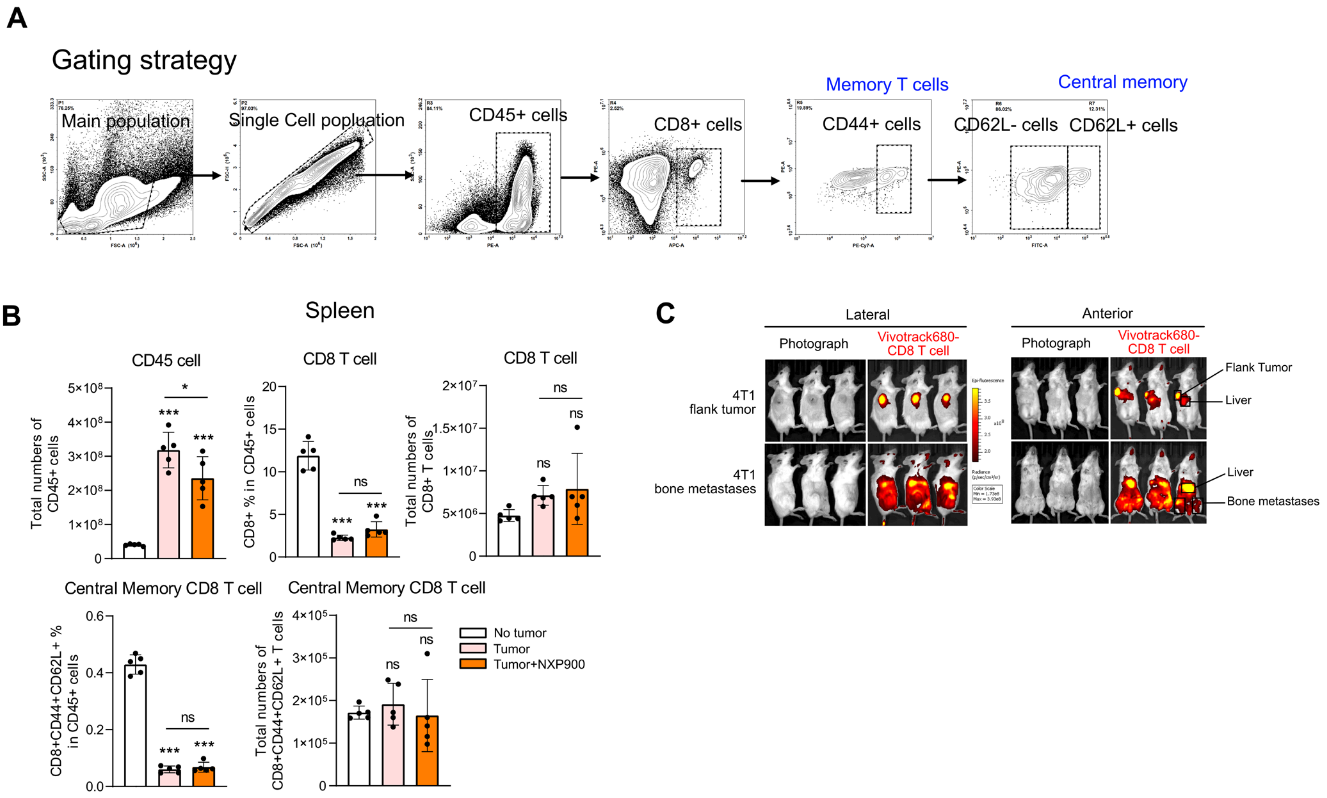

Figure S13, related to Figure 6. Induction of CD8⁺ memory T-cell formation by NXP900 treatment.

(A) Gating strategy used for the analysis shown in Figure 6I and 6J. (B) Quantification of central memory $CD8^+$ T cells in the spleen following NXP900 treatment. Absolute immune cell numbers represent counts from a whole spleen. The same samples were used as in Figure 6I. N = 5/group. (C) Validation of tumor-targeting capacity of isolated central memory $CD8^+$ T cells. Purified CD8 T cells were labeled with VivoTrack680 and tracked *in vivo* after transfer. All data were presented as mean with SD. Statistical significance of the differences was determined by one-way ANOVA followed by Tukey's test. *$p < 0.05$, **$p < 0.01$, ***$p < 0.001$, significant difference between indicated points.

Table S1. The used cell lines and their respective culture methods.

DMEM: Dulbecco's modified Eagle's medium

FBS: Fetal bovine serum, Thermo Fisher Scientific, Waltham, MA, USA

P/S: penicillin/streptomycin, Hyclone, Logan, UT, USA

Cell line	Culture Medium
**4T1**	RPMI-1640 medium + 10% FBS + 1% P/S
**4T1-luc**	RPMI-1640 medium + 10% FBS + 1% P/S
**B16F10-luc**	High glucose DMEM medium + 10% FBS + 1% P/S
**HEK-293T**	High glucose DMEM medium + 10% FBS + 1% P/S
**MCF-7**	High glucose DMEM medium + 10% FBS + 1% P/S
**MDA-MB-231**	High glucose DMEM medium + 10% FBS + 1% P/S
**PC-3**	RPMI-1640 medium + 10% FBS + 1% P/S
**MG-63**	High glucose DMEM medium + 10% FBS + 1% P/S
**U2-OS**	High glucose DMEM medium + 10% FBS + 1% P/S

Table S2. The used antibodies.

Antibodies for western blotting

Name	Company	Cat No.
**COX-2**	Abcam (Cambridge, UK)	Ab15191
**Arginase-1**	Santa Cruz Biotechnology (Dallas, TX, USA)	sc-271430
**PD-L1 (human)**	Cell Signaling Technology (Danvers, MA, USA)	13684T
**PD-L1 (mouse)**	Cell Signaling Technology	60475T
**GAPDH**	Merck Millipore (Burlington, MA, USA)	CB1001
**Phospho-SRC Y416**	Cell Signaling Technology	2101s
**Phospho-SRC Y530**	Cell Signaling Technology	2105s
**SRC**	Cell Signaling Technology	2108s
**p-FAK**	Cell Signaling Technology	3283s
**FAK**	Merck Millipore	05-537
**p-Paxillin**	Cell Signaling Technology	69363s
**Paxillin**	Cell Signaling Technology	2542s
**MMP-9**	Cell Signaling Technology	24317T
**N-Cadherin**	BD Biosciences (Franklin Lakes, NJ, USA)	610920
**E-Cadherin**	BD Biosciences	610181
**α-Actinin**	Proteintech (Rosemont, IL, USA)	66895-1-Ig
**Cortactin**	Cell Signaling Technology	3503s
**Phospho-Cortactin**	Cell Signaling Technology	4569s
**LIMK1**	Proteintech	67974-1-Ig
**p-LIMK1/2**	Merck Millipore	07-850
**Cofilin**	Santa Cruz Biotechnology	sc-376476
**p-Cofilin**	Santa Cruz Biotechnology	sc-271921
**Cathepsin K**	Santa Cruz Biotechnology	sc-48353

**TRAP/ACP5**	**Santa Cruz Biotechnology**	**sc-376875**
**Cyclin B1**	Santa Cruz Biotechnology	sc-245
**Cyclin D1**	Cell Signaling Technology	2922s
**p-PI3K**	Cell Signaling Technology	17366T
**PI3K**	Cell Signaling Technology	4257T
**p-AKT**	Cell Signaling Technology	9271s
**AKT**	Cell Signaling Technology	9272s
**p-MEK**	Cell Signaling Technology	9154s
**MEK**	Cell Signaling Technology	8727s
**p-ERK**	Cell Signaling Technology	9101s
**ERK**	Cell Signaling Technology	9102s
**p-JNK**	Cell Signaling Technology	9251s
**JNK**	Cell Signaling Technology	9252T
**p-c-Jun**	Cell Signaling Technology	9261s
**c-Jun**	Santa Cruz Biotechnology	sc-1694
**p-NFκB**	Cell Signaling Technology	3033s
**NFκB**	Santa Cruz Biotechnology	sc-8008
**p-STAT3**	Cell Signaling Technology	9131s
**STAT3**	Cell Signaling Technology	4904s
**p- IκB-α**	Cell Signaling Technology	2859s
**IκB-α**	Cell Signaling Technology	9242s
**HRP-linked anti-rabbit IgG**	Cell Signaling Technology	7074s
**HRP-linked anti-mouse IgG**	Cell Signaling Technology	7076s

Antibodies for flow cytometry

Name	Company	Cat No.
**PE anti-mouse MMP9**	Santa Cruz Biotechnology	sc-13520 PE
**APC anti-human PD-L1**	Biolegend (San Diego, CA, USA)	329707
**APC anti-mouse PD-L1**		124311
**PE-Cy7 anti-mouse F4/80**		123114
**APC-Cy7 anti-mouse CD86**		105030
**PE anti-mouse CD206**		141705
**PE anti-mouse CD45**		103105
**APC anti-mouse CD8**		100711
**PE-Cy7 anti-mouse CD44**		103029
**FITC anti-mouse CD62L**		104405

Table S3. The used drugs.

Name	Company	Cat No.
**NXP900 (eCF506)**	TargetMol<br>(Boston, MA, USA)	T8499
**Dasatinib**	MedChemExpress<br>(Monmouth Junction, NJ, USA)	HY-10181
**Saracatinib**	MedChemExpress	HY-10234
**Cytochalasin D**	Thermo Fisher Scientific<br>(Waltham, MA, USA)	PHZ1063
**Picropodophyllin**	MedChemExpress	HY-15494
**Mouse IGF-1 recombinant protein**	Thermo Fisher Scientific	250-19-50UG
**Gemcitabine**	MedChemExpress	HY-17026
**Zoledronate**	MedChemExpress	HY-13777
**Anti-mouse PD-1 antibody**	BioXCell<br>(Lebanon, NH, USA)	BE0146
