# Supplementary material for: Inhibition of SRC prevents bone metastasis of breast cancer by blocking metastatic cell motility and bone directionality
Source: Theranostics. 2026 May 18;16(12):6928–47. doi: 10.7150/thno.130647 (PMC13232475; doi:10.7150/thno.130647)

Fig. 2C

3 Biological Replicates
MDA-MB-231

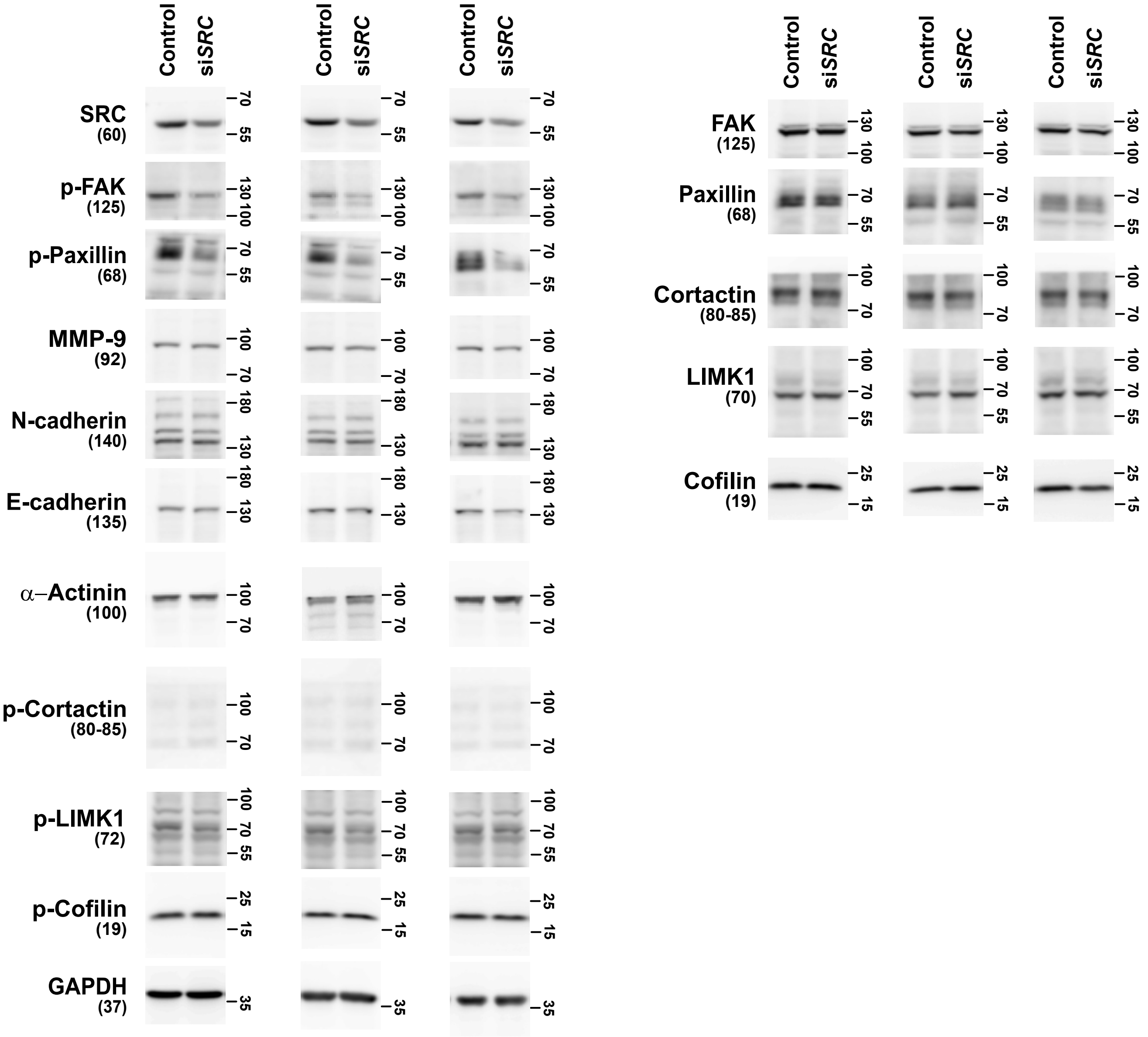

Fig. 2D

3 Biological Replicates
MDA-MB-231

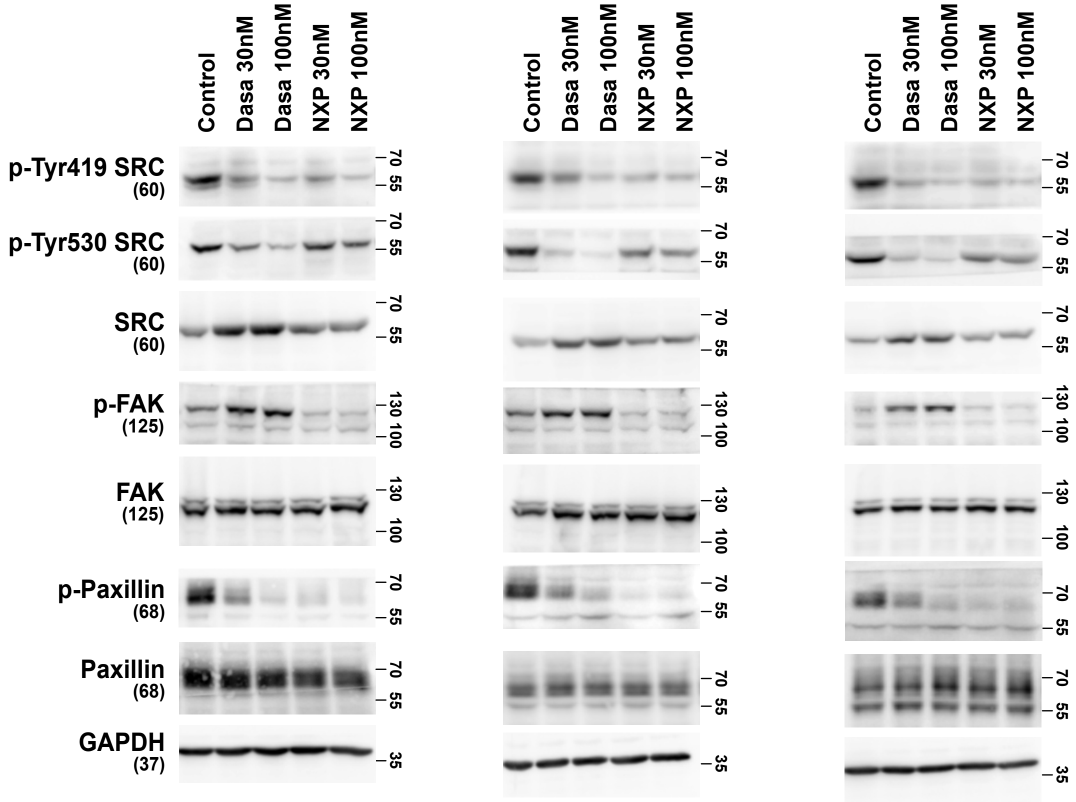

3 Biological Replicates
4T1

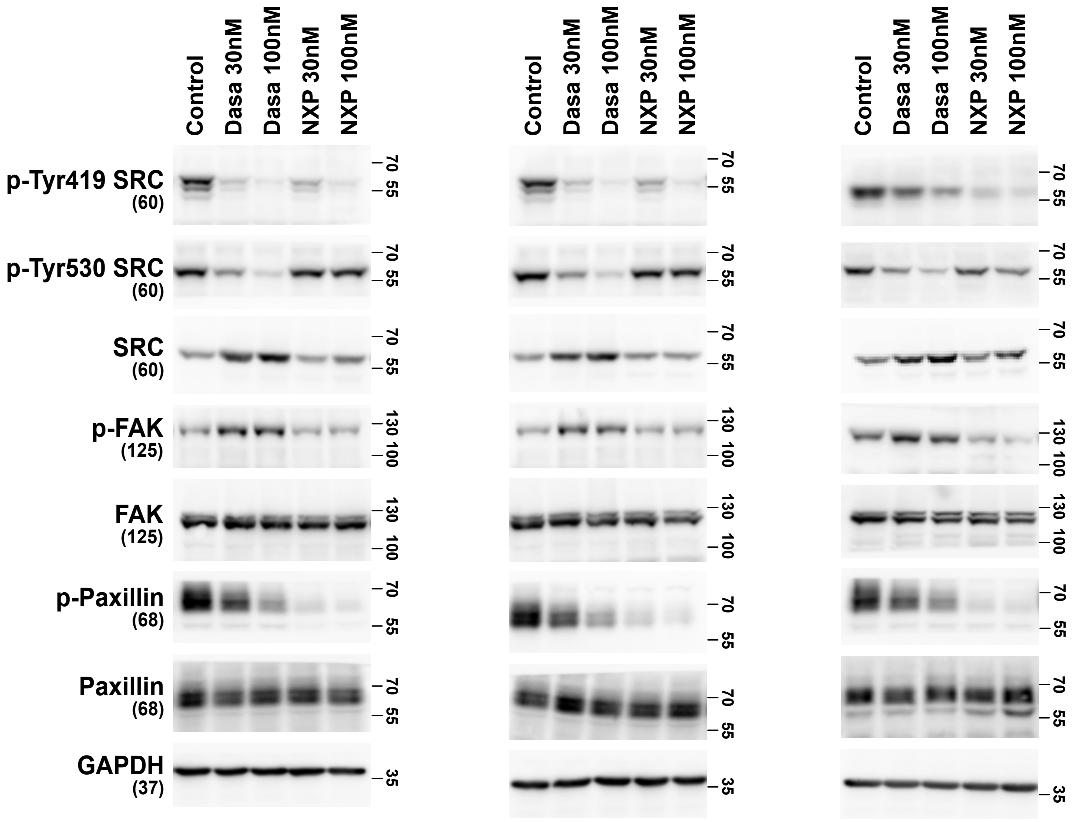

Fig. S2E

3 Biological Replicates
PC-3

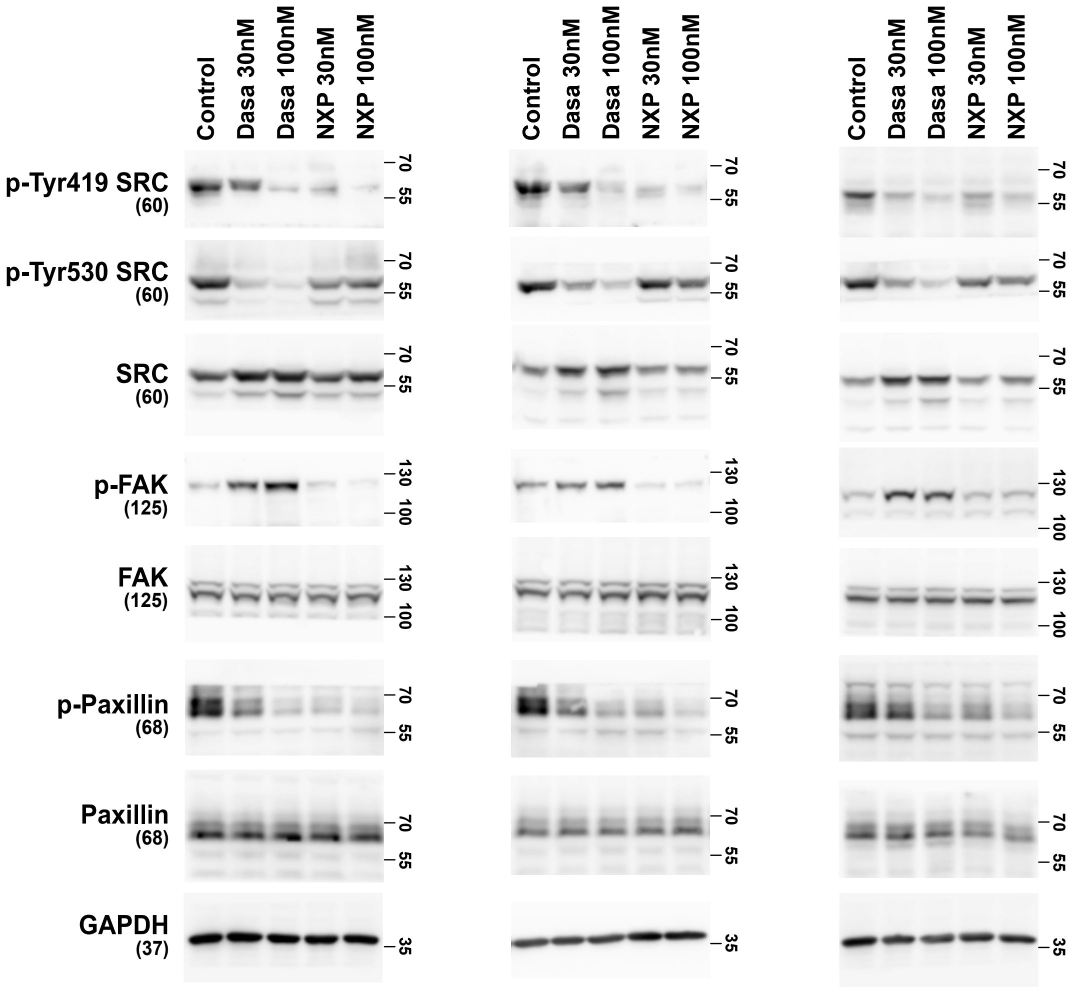

Fig. S2F

3 Biological Replicates

MG-63

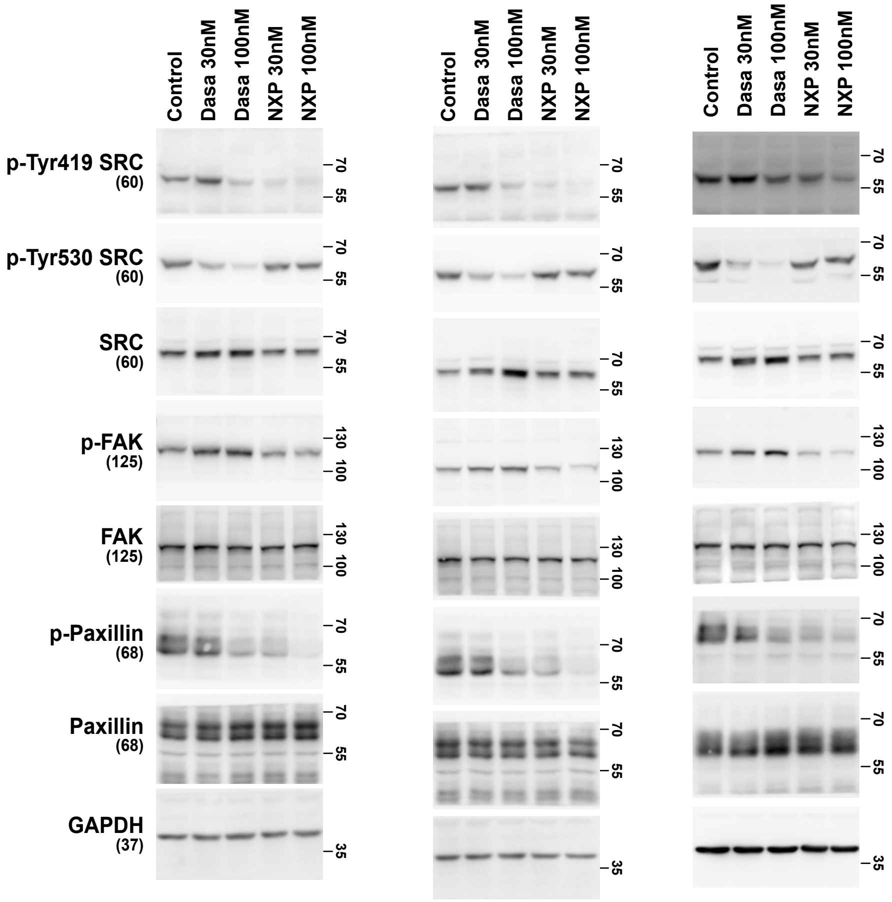

3 Biological Replicates

U2-OS

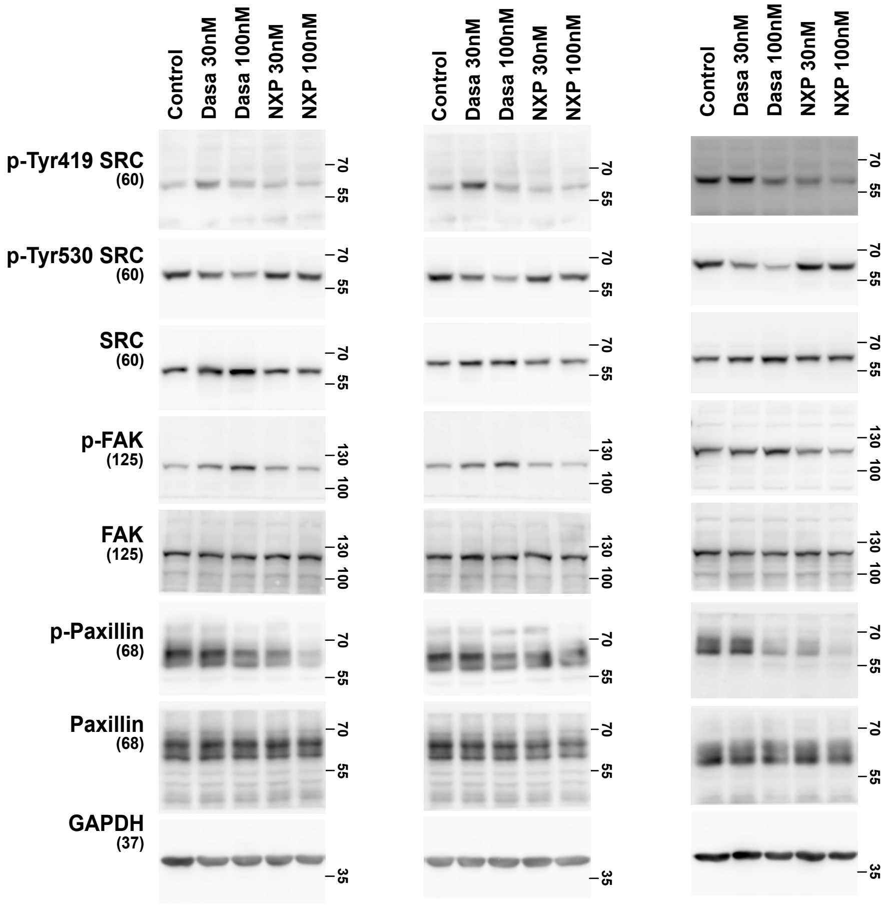

Fig. S3B

3 Biological Replicates

MCF7

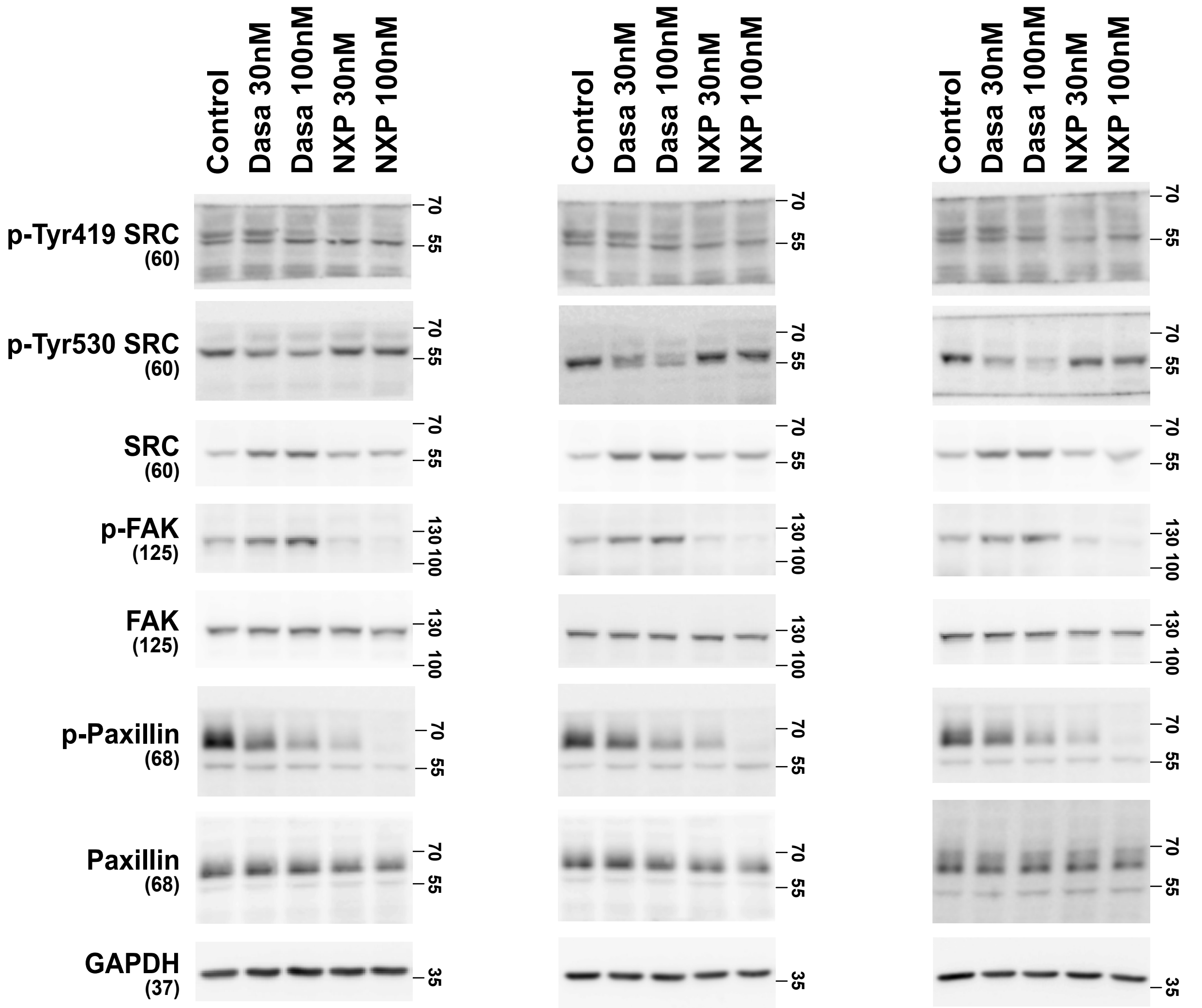

Fig. S4C

3 Biological Replicates

MDA-MB-231

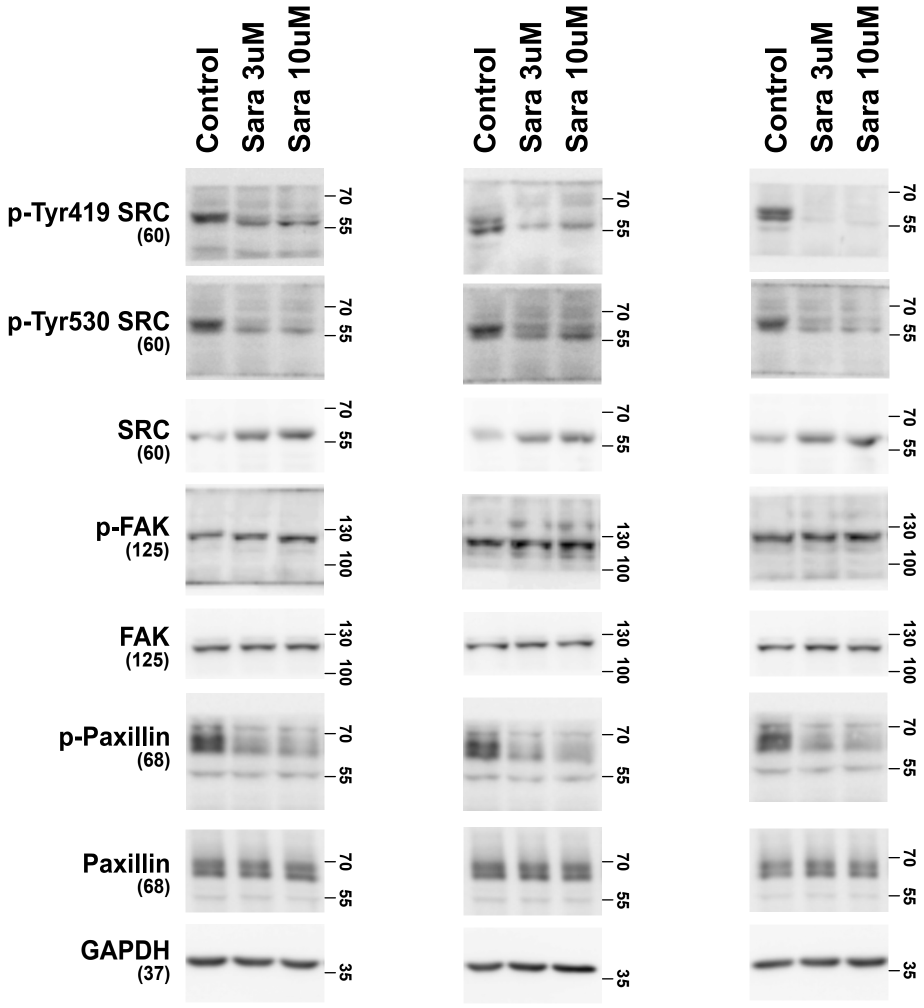

Fig. S5C

3 Biological Replicates

MDA-MB-231

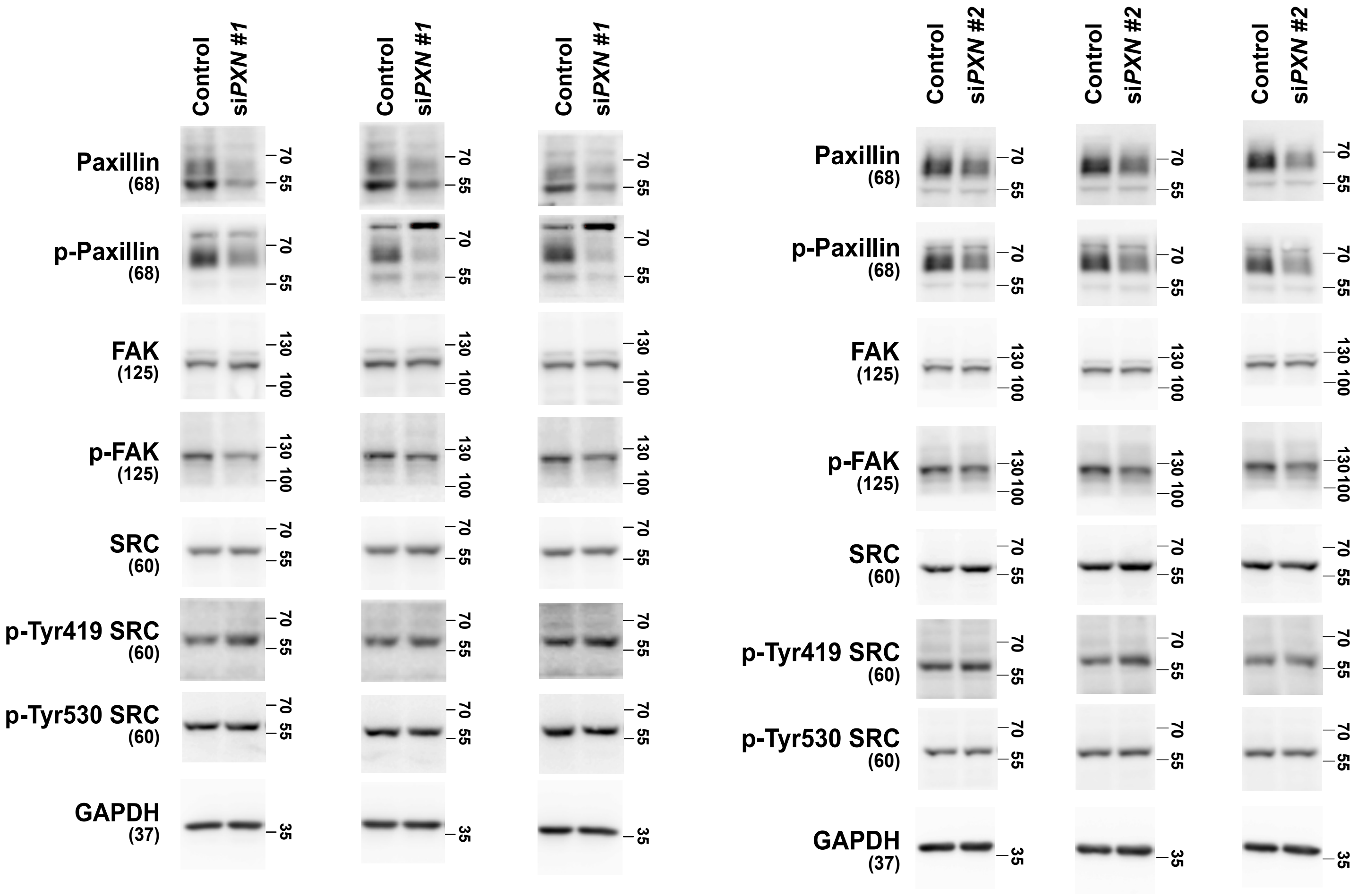

Fig. S6C

3 Biological Replicates

MDA-MB-231

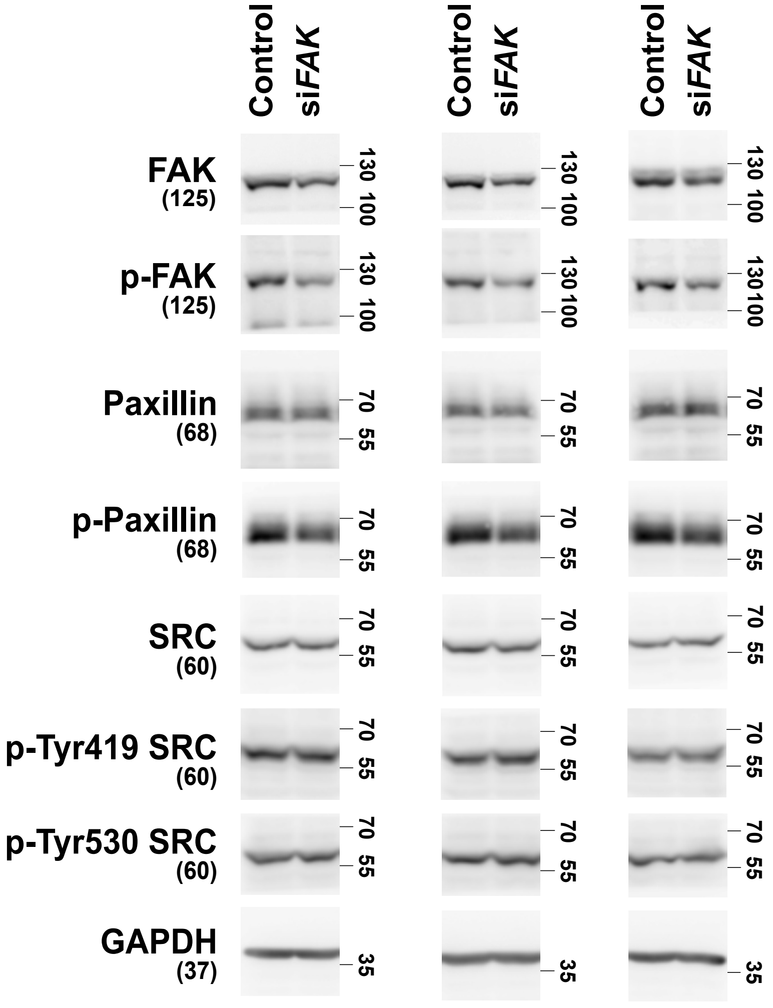

Fig. 3D

4 Biological Replicates

MDA-MB-231

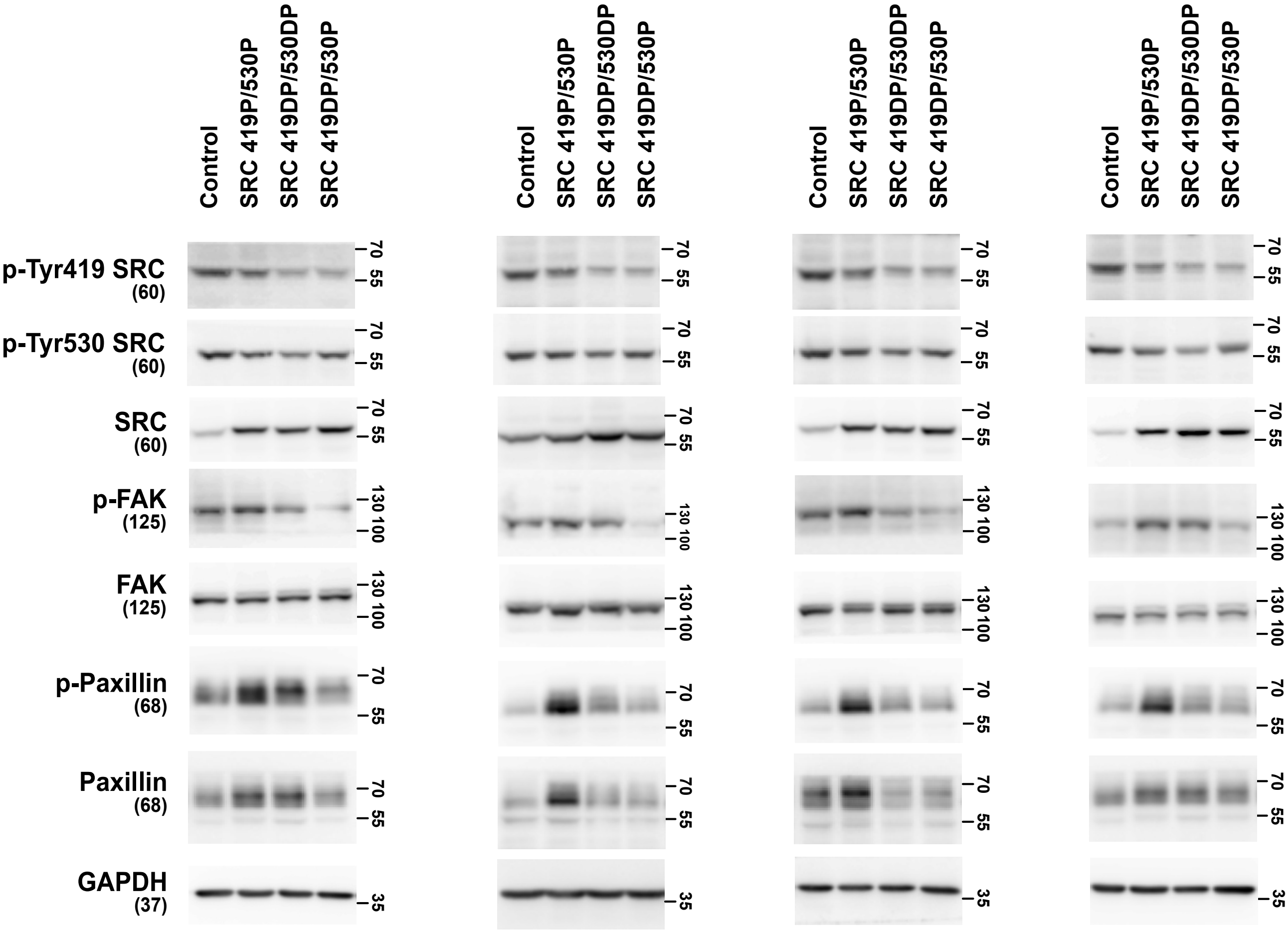

Fig. S7A

3 Biological Replicates

MDA-MB-231

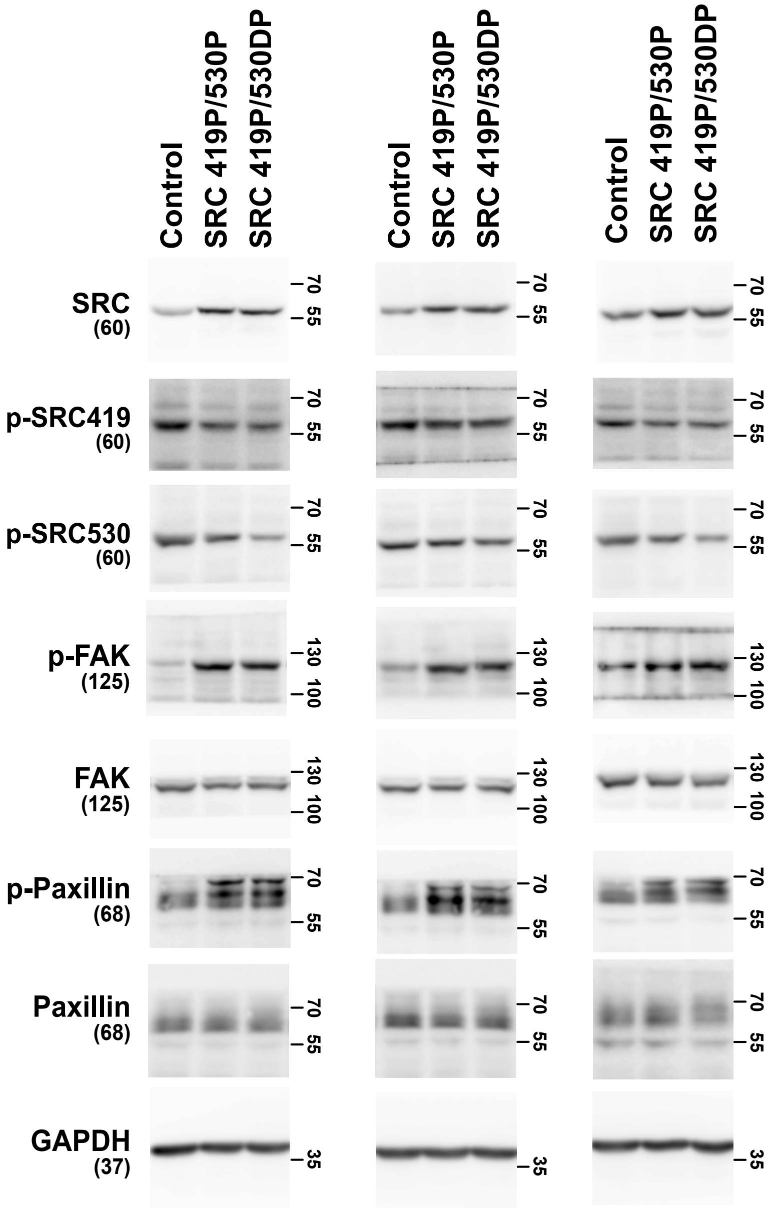

Fig. 4B

3 Biological Replicates

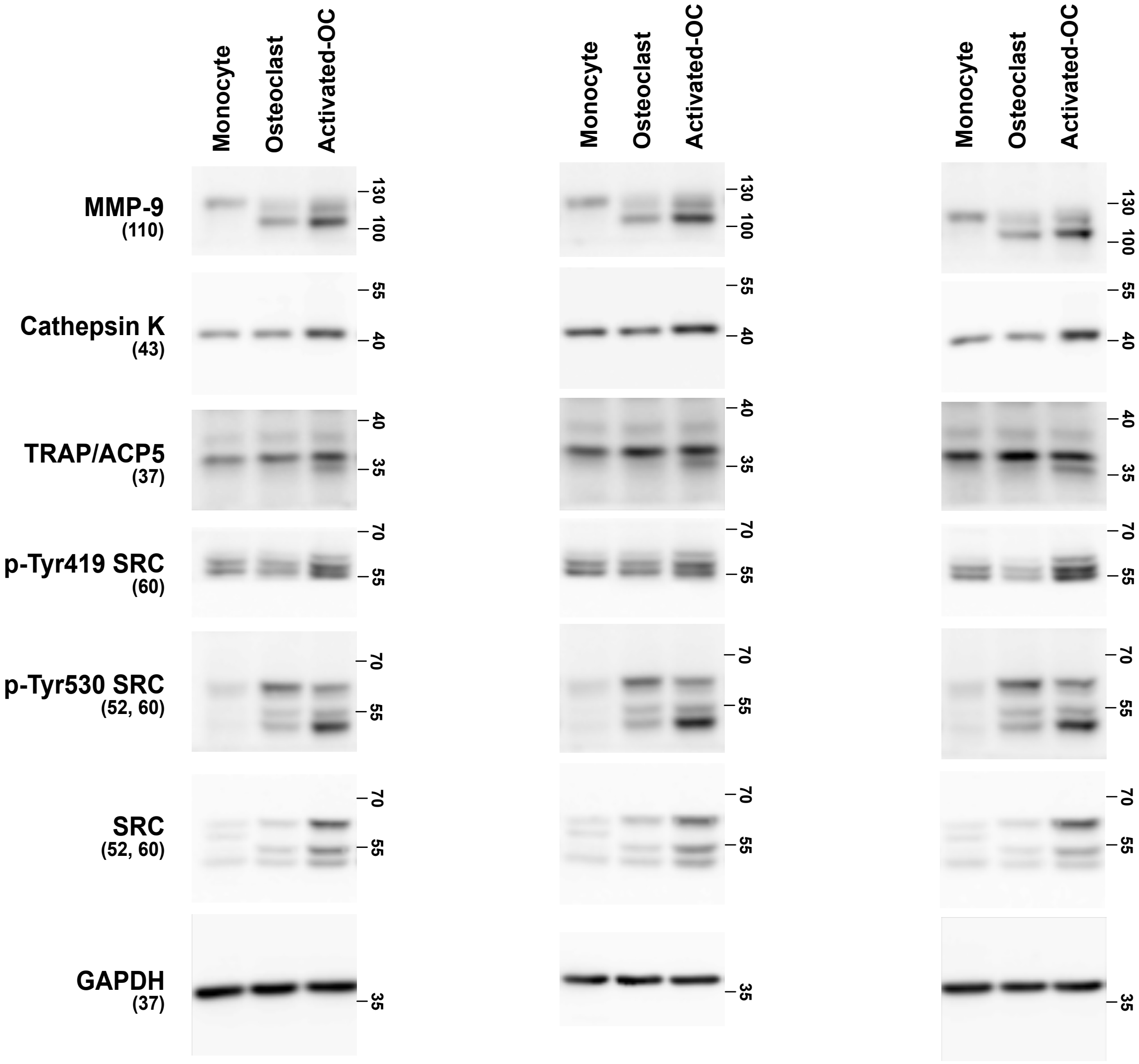

Fig. 4H

3 Biological Replicates

4T1

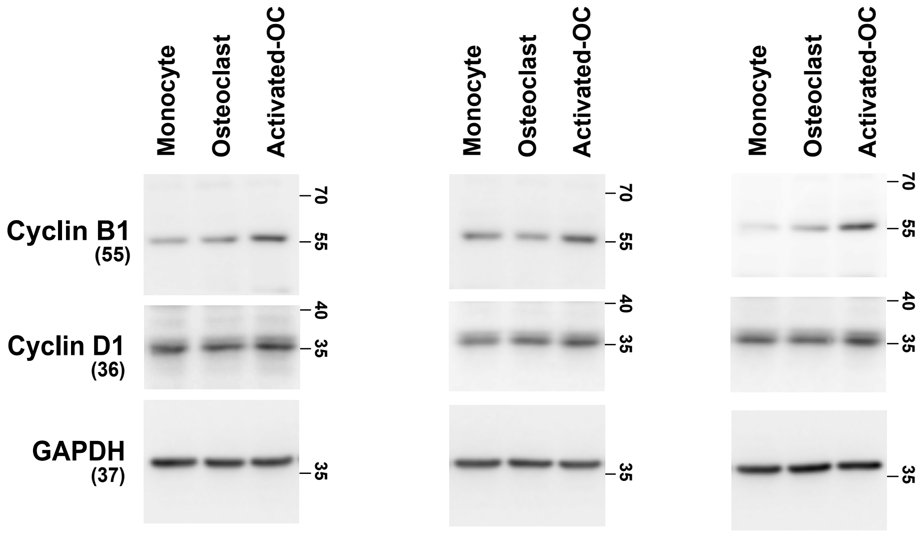

Fig. 4L

3 Biological Replicates

4T1

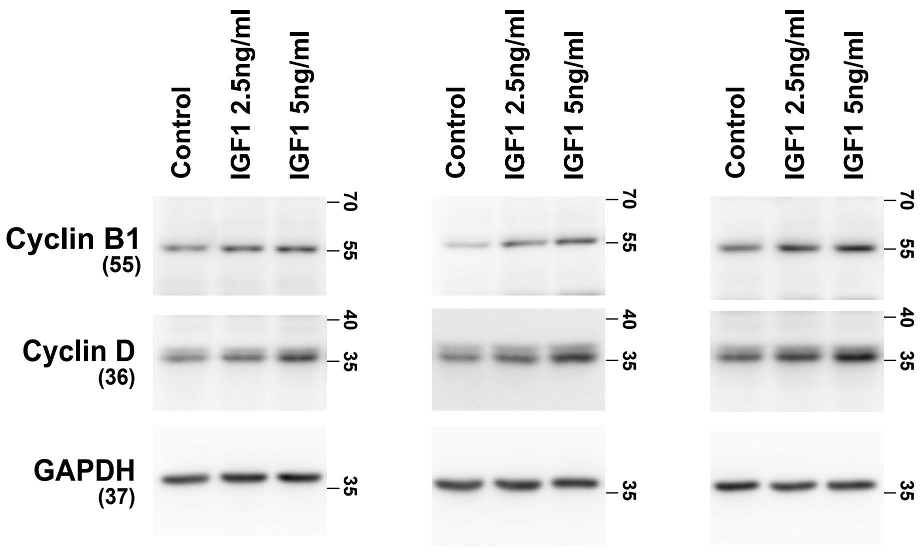

Fig. 4M

3 Biological Replicates
Activated-Osteoclast

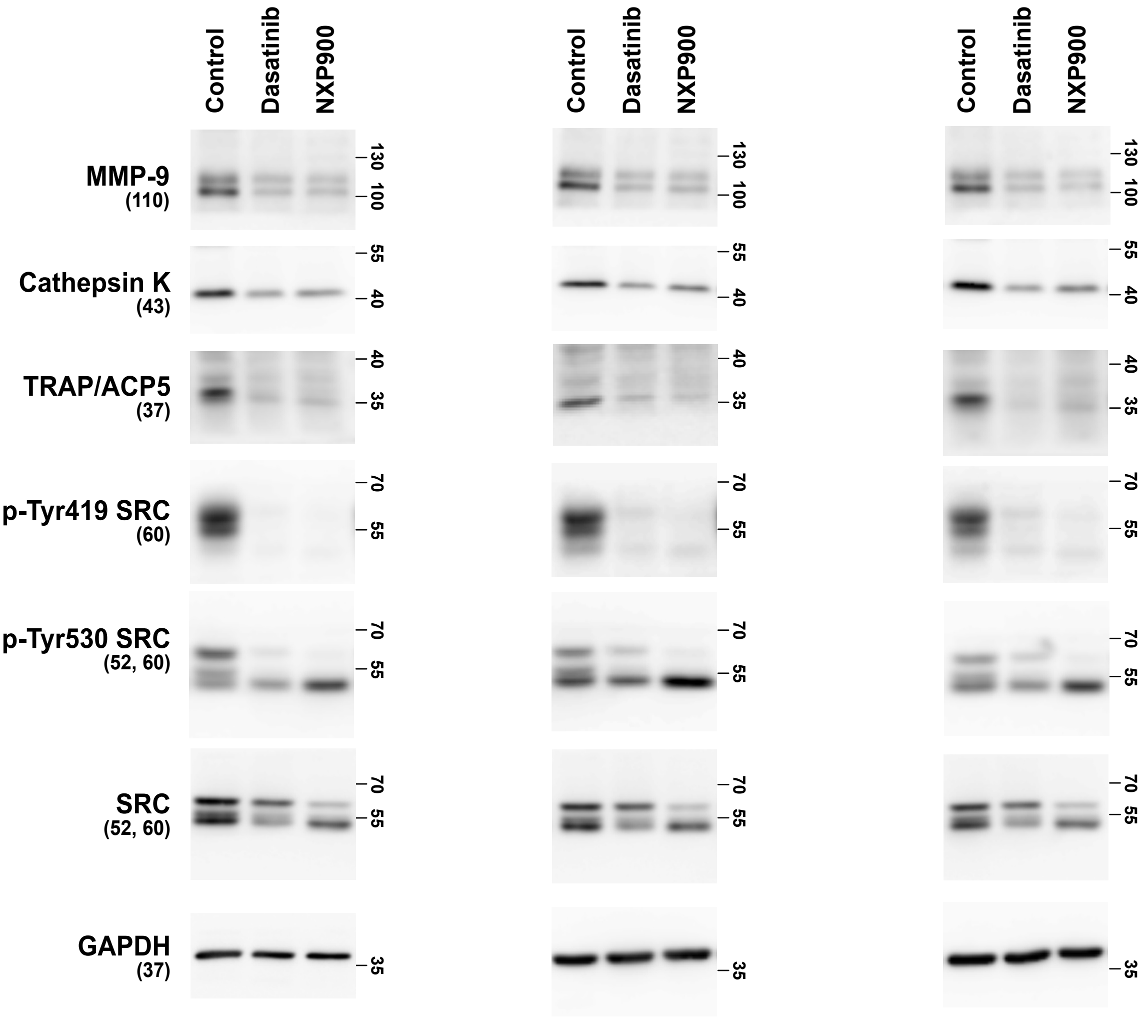

Fig. S9A

3 Biological Replicates

4T1

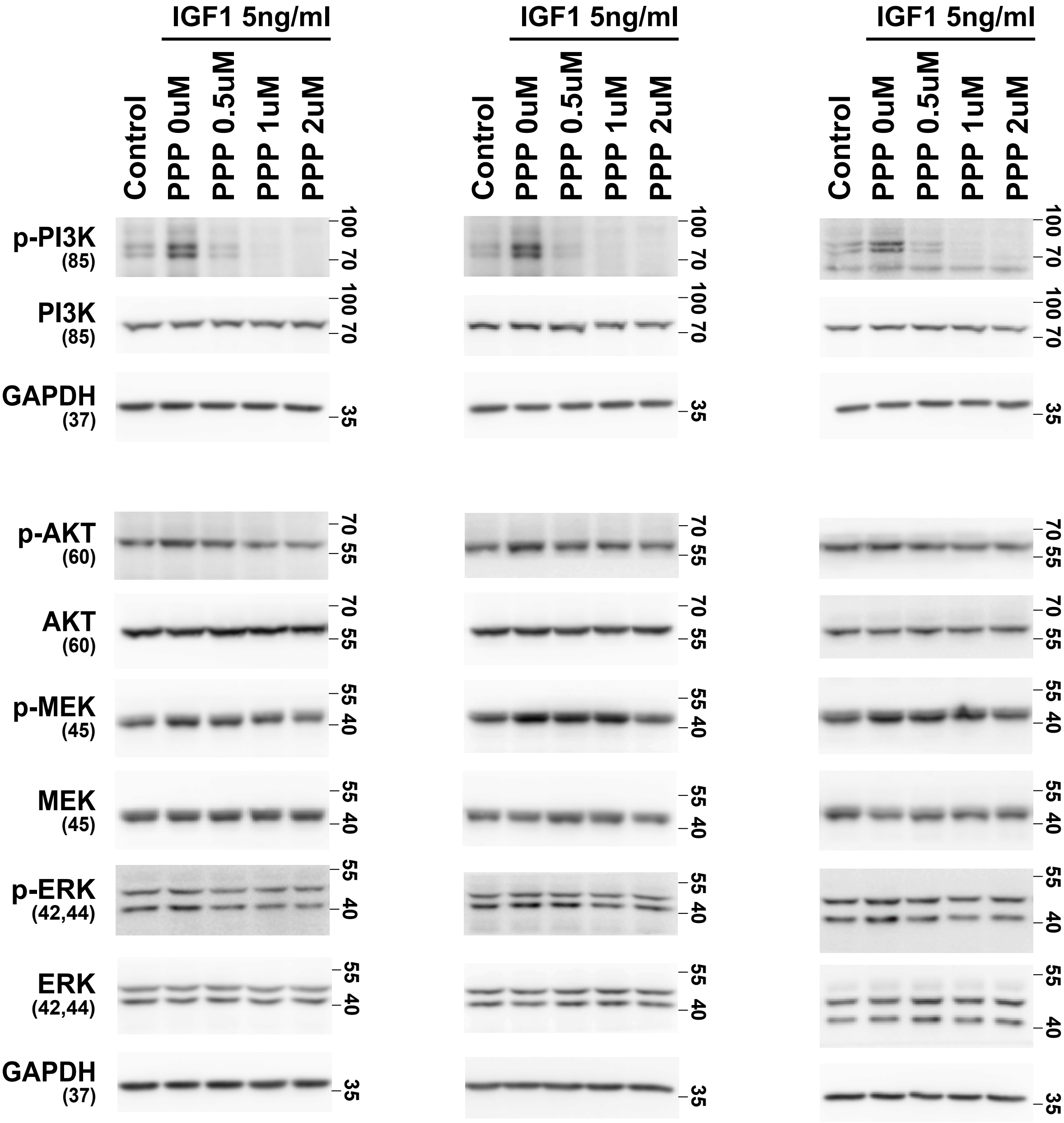

Fig. S10A

3 Biological Replicates

Activated-Osteoclast

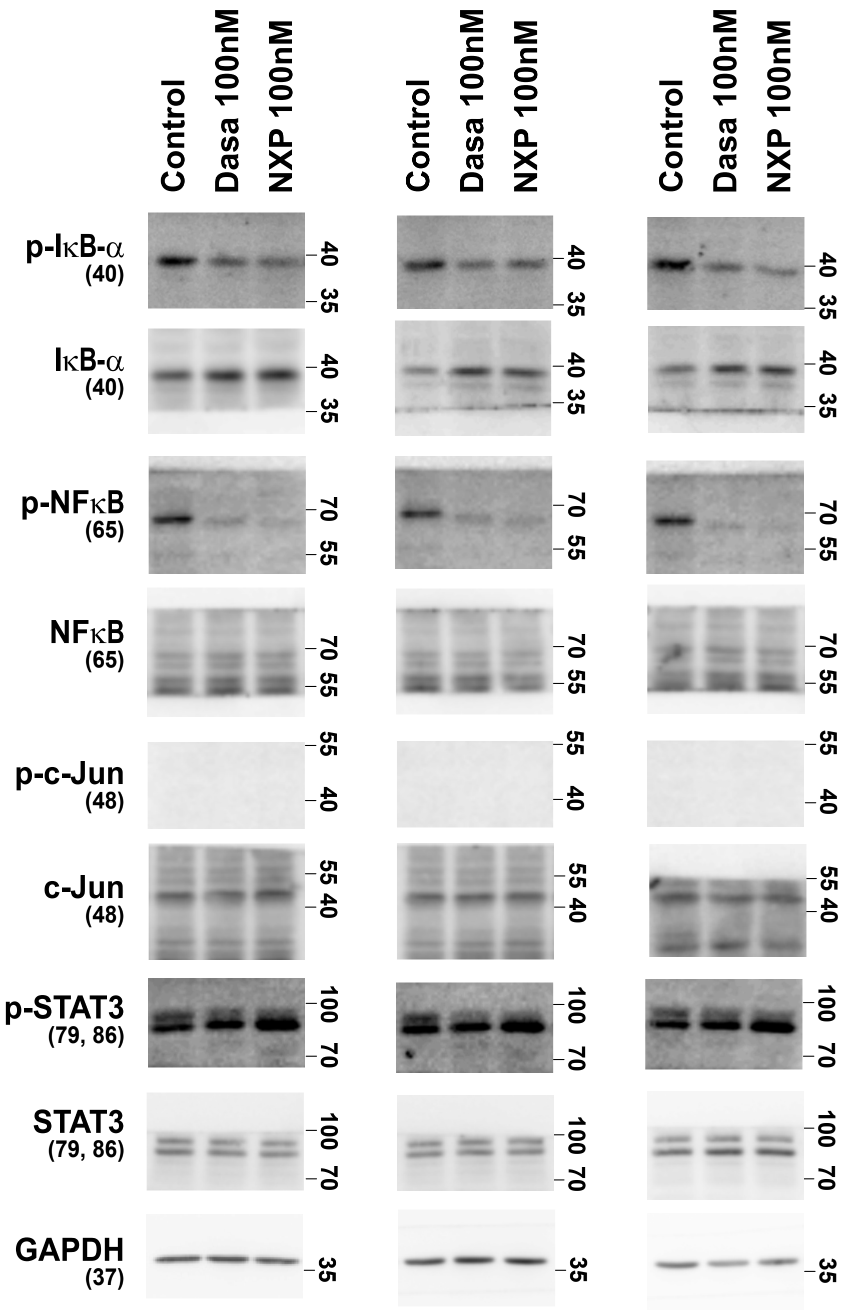

Fig. 6C

3 Biological Replicates
MDA-MB-231

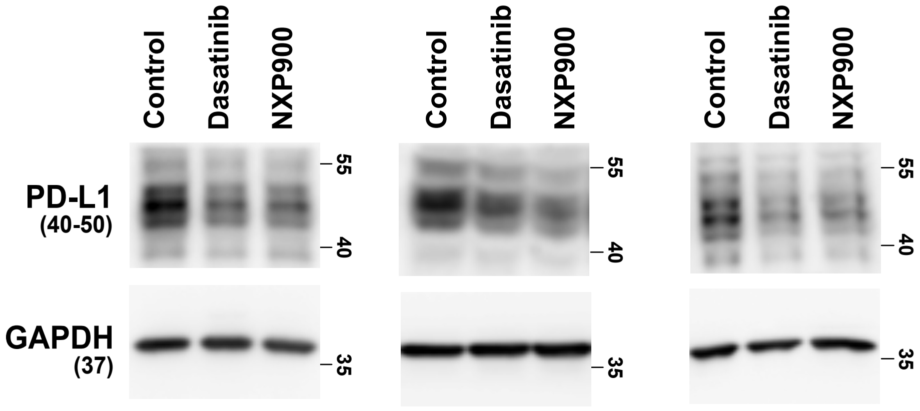

3 Biological Replicates
4T1

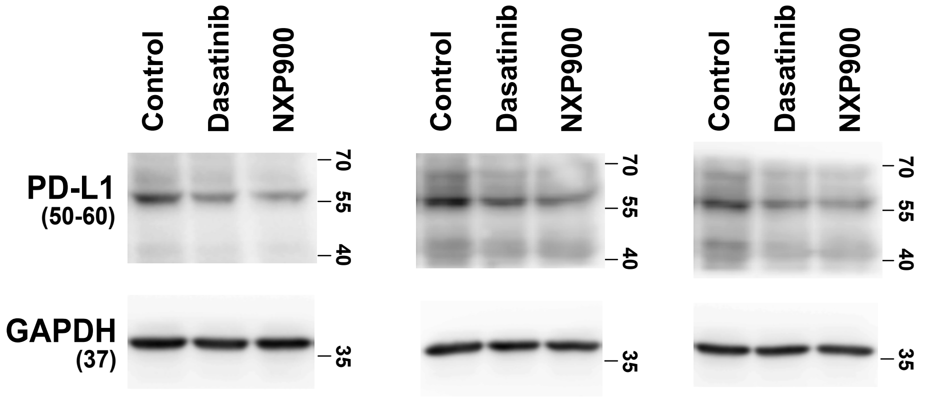

Fig. 6E

3 Biological Replicates

BMDM

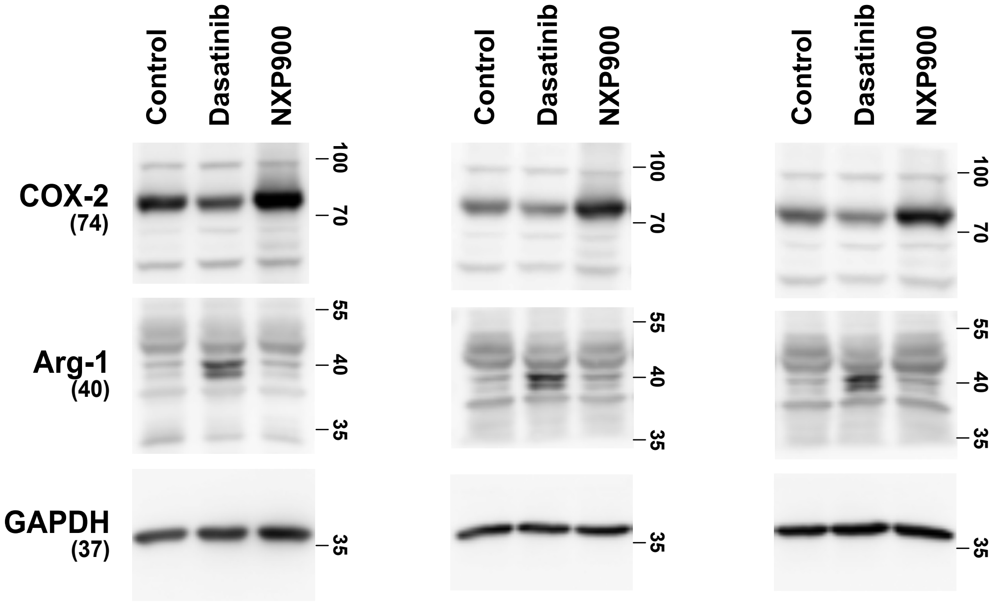

Fig. S12A

3 Biological Replicates

MDA-MB-231

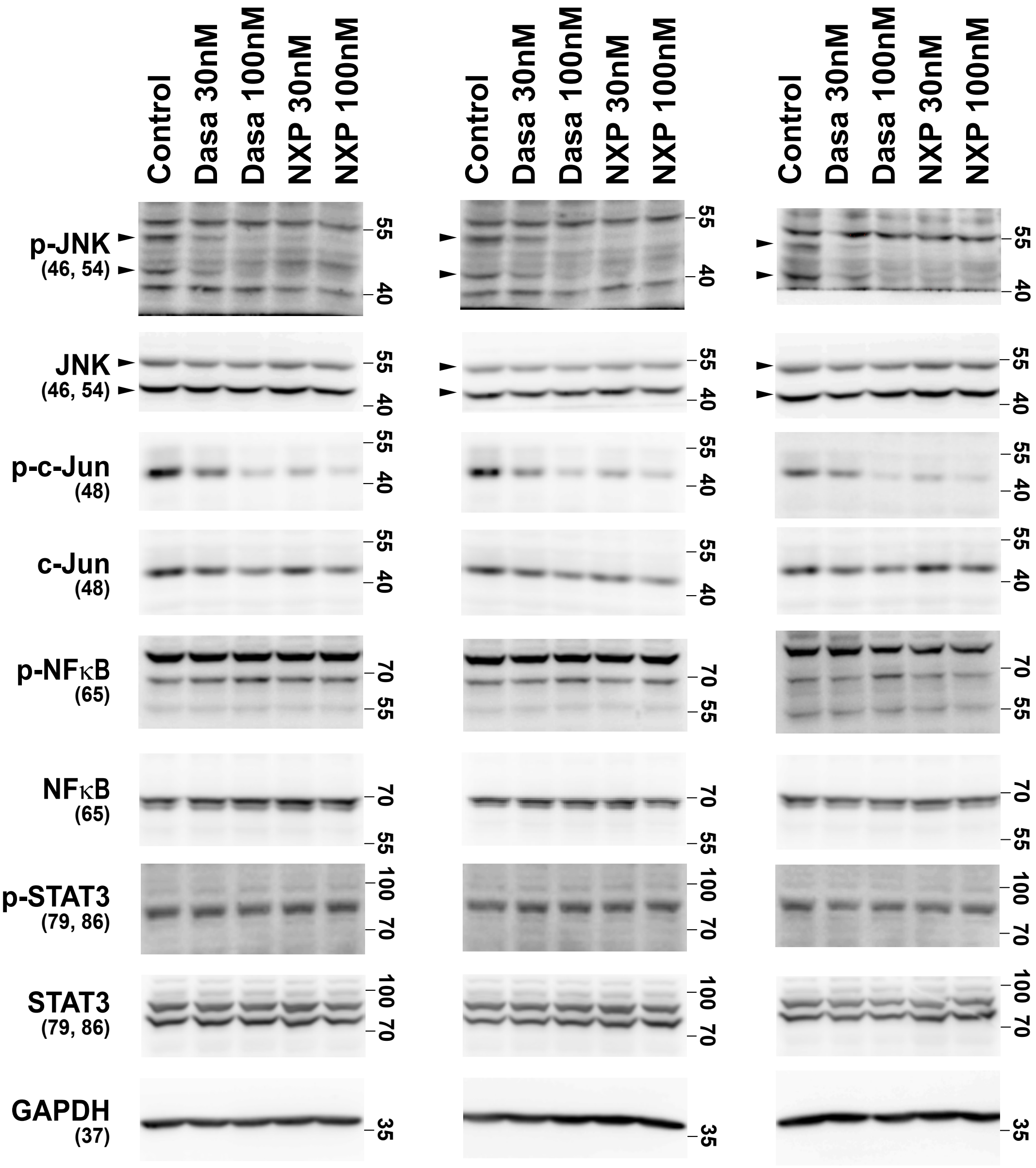

Supplement: Supplementary file 2 — Supplementary original blots. [file thnov16p6928s2.pdf]
